# Chloroquine and amodiaquine enhance AMPK phosphorylation and improve mitochondrial fragmentation in diabetic tubulopathy

**DOI:** 10.1038/s41598-018-26858-8

**Published:** 2018-06-08

**Authors:** Hye Yun Jeong, Jun Mo Kang, Hak Hoon Jun, Dong-Jin Kim, Seon Hwa Park, Min Ji Sung, Jin Hyung Heo, Dong Ho Yang, Sang Ho Lee, So-Young Lee

**Affiliations:** 1 Division of Nephrology, Department of Internal Medicine, CHA Bundang Medical Center, CHA University, Seongnam, South Korea; 2 Department of Surgery, CHA Bundang Medical Center, CHA University, Seongnam, South Korea; 3 Division of Nephrology, Department of Internal Medicine, Kyung Hee University Hospital at Gangdong, Kyung Hee University, Seoul, South Korea; 4 Department of Pathology, CHA Bundang Medical Center, CHA University, Seongnam, South Korea

## Abstract

We investigated the effects of chloroquine (CQ) and amodiaquine (AQ) on AMPK phosphorylation in renal tubular cells in a diabetic environment *in vivo* and *in vitro*. We also examined whether CQ- or AQ-mediated AMPK activity restoration attenuated diabetic tubulopathy by normalizing mitochondrial fragmentation. Human renal proximal epithelial cells (HKC8) were incubated in high-glucose conditions. Diabetes was induced with streptozotocin in male C57/BL6J mice. Treatment with CQ or AQ abolished high-glucose-induced phospho-AMPK and phosph-PGC1α down-regulation in HKC8 cells. Improvements in functional mitochondrial mass and balanced fusion/fission protein expression were observed in HKC8 cells after treatment with CQ or AQ in high-glucose conditions. Moreover, decreased mitochondrial ROS production and reduced apoptotic and fibrotic protein expression were noted in HKC8 cells after treatment with CQ or AQ, even in high-glucose conditions. CQ and AQ treatment effectively mitigated albuminuria and renal histopathologic changes and increased AMPK activity in the kidneys of diabetic mice. Electron microscopy analysis showed that mitochondrial fragmentation was decreased, and 8-OHdG content was low in the renal tubular cells of the CQ and AQ treatment groups compared with those of the diabetic control group. Our results suggest that CQ and AQ may be useful treatments for patients with diabetic kidney disease.

## Introduction

Diabetic kidney disease (DKD) is a common complication of diabetes and is a prime indication for dialysis^1^. Unfortunately, there are no definitive treatment modalities capable of inhibiting the progression of kidney dysfunction; however, blood glucose and blood pressure control and angiotensin converting enzyme inhibitors have been used for a long time^2^. The activity of 5′ AMP-activated protein kinase (AMPK), the major energy-sensing enzyme, was recently observed to be reduced in the kidneys of both diabetic mice and humans^3^. Several previous studies, including ours, showed that AMPK activators attenuate diabetic nephropathy, resulting in decreased albuminuria in diabetic mice^3,4^.

Chloroquine and amodiaquine are members of the drug class 4-aminoquinoline and have been used as antimalaric drugs worldwide^5,6^. Chloroquine has also been considered an essential therapy in patients with systemic lupus erythematosus (SLE)^7^. Withdrawal of amodiaquine treatment is related to lupus flares within 1–3 months of medication cessation^8^. Both drugs have been suggested to have anti-inflammatory, immune-suppressive and photoprotective effects, but the mechanisms by which these agents act on SLE are unclear^9–13^. Interestingly, a recent study reported that chloroquine—similar to resveratrol, a known AMPK activator—increased AMPK phosphorylation in myotube cells^14^.

In the present study, we demonstrated that chloroquine and amodiaquine attenuate diabetic tubulopathy by decreasing mitochondrial damage by enhancing AMPK phosphorylation in human renal proximal tubular cells (hRPTCs) subjected to high-glucose environment and streptozotocin (STZ)-induced diabetic mouse kidneys.

## Results

### Chloroquine and amodiaquine increase AMPKα and peroxisome proliferator-activated receptor gamma coactivator 1-alpha (PGC1α) phosphorylation in RPTCs subjected to high-glucose conditions

**Figure 1:**
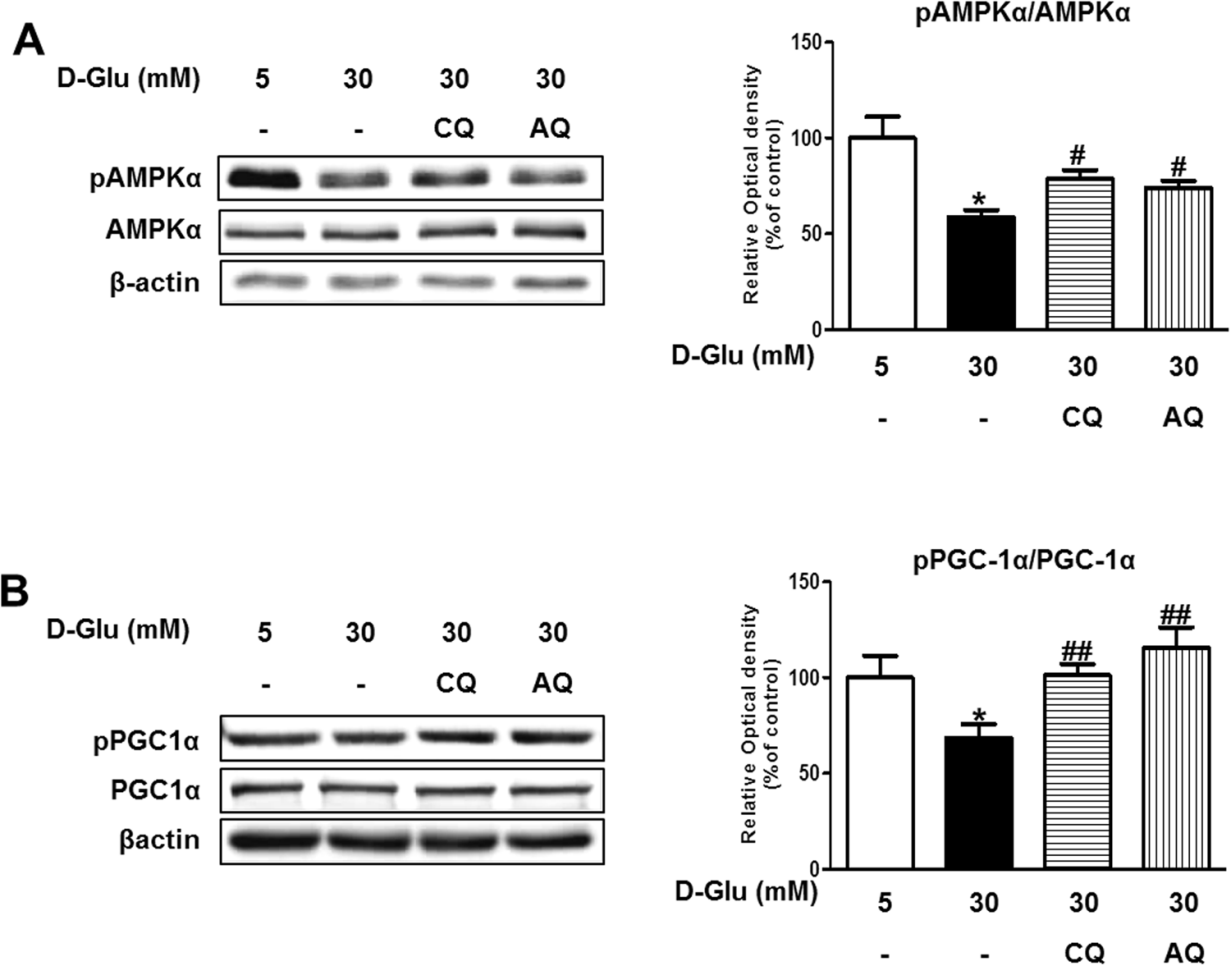
Chloroquine (CQ) or amodiaquine (AQ) restored pAMPKα and pPGC1α expression in hRPTCs under high-glucose (HG) conditions. 3 × 10^5^ cells were seeded in a 60 mm dish with 5–30 mM D-glucose and cultured for 6 hours. To explore the effects of the drugs on hRPTCs subjected to HG conditions, CQ (200 μM) or AQ (10 μM) were treated for 1 hour before changing culture medium into 30 mM D-glucose. Cell extracts were immunoblotted with anti-phospho-AMPKα, anti-AMPKα, anti-phospho-PGC1α, anti-PGC1α and anti-βactin antibody. (**A**) Western blot analyses revealed that AMPKα phosphorylation was increased in hRPTCs treated with CQ or AQ under HG conditions. Band intensities representing phospho-AMPKα and AMPKα expression levels were converted into densitometry using ImageJ software in the ratio of phospho-AMPKα to AMPKα. Results are means ± SEM. for experiments in triplicate. (**B**) Representative immunoblot data showed that PGC1α phosphorylation was also enhanced in hRPTCs after treatment with CQ or AQ under HG conditions. Band intensities representing phospho-PGC1α and PGC1α expression levels were converted into densitometry using ImageJ software in the ratio of phospho-PGC1α to PGC1α. Results are means ± SEM. for experiments in triplicate. (*p < 0.05 vs 5 mM, ^#^p < 0.05, ^##^p < 0.01 vs 30 mM).

First, we investigated whether chloroquine or amodiaquine effectively augments AMPK phosphorylation in hRPTCs cultured under high-glucose conditions. AMPK and PGC1α phosphorylation was reduced under high-glucose conditions; however, treatment with chloroquine or amodiaquine abolished high-glucose induced phospho-AMPKα and phospho-PGC1α down-regulation in hRPTCs (Fig. 1, and see Supplementary Fig. S1). These effects were dose-dependent, as shown in Supplementary Fig. S2. Treatment with high-dose amodiaquine at a concentration higher than 100 μM induced cytotoxicity. Our observation that AMPKα and PGC1α activity was suppressed in renal tubular cells subjected to high-glucose conditions is consistent with the findings of previous reports^4^.

**Figure 2:**
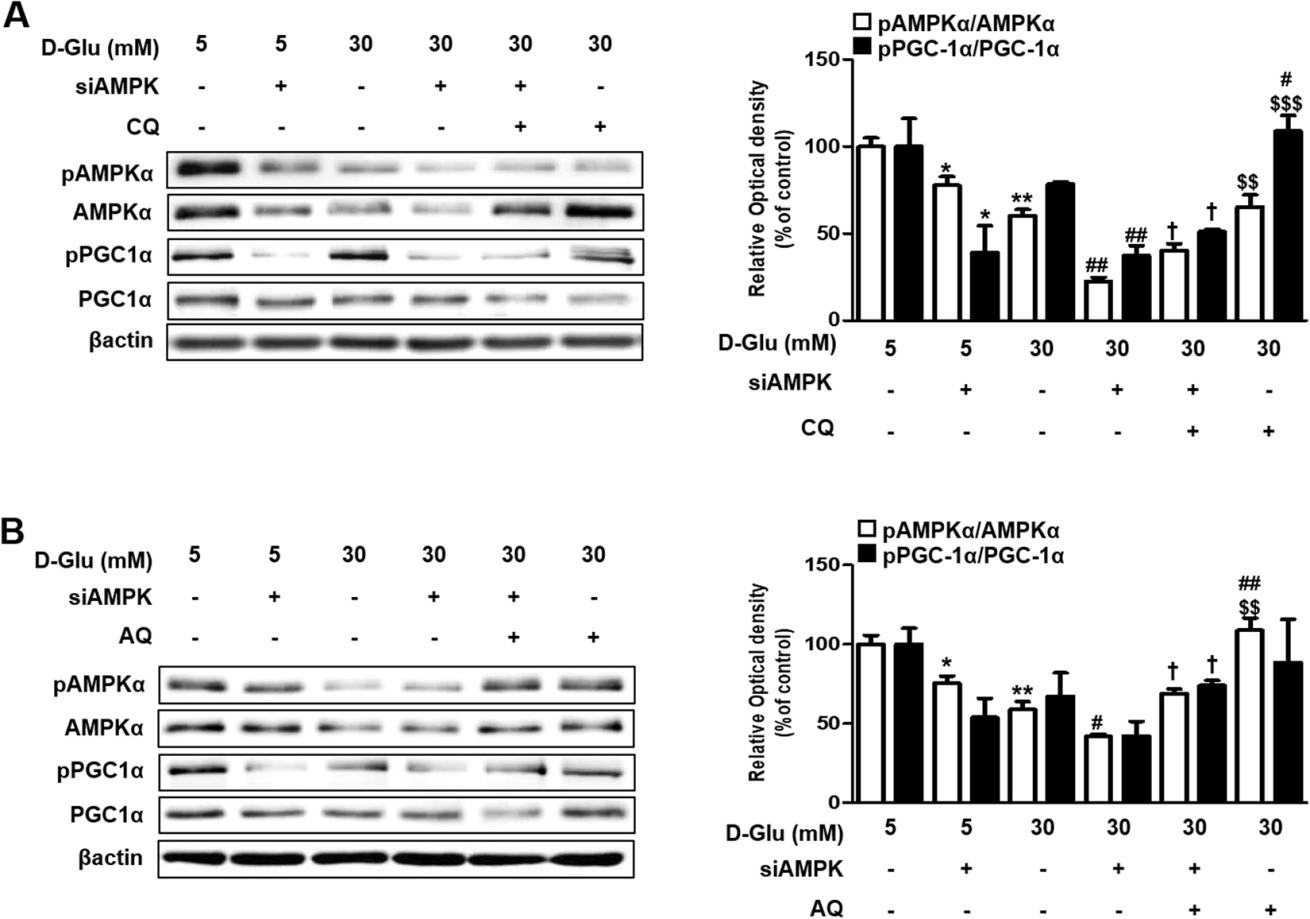
Effect of siAMPK on CQ- or AQ- induced PGC1α phosphorylation. HKC-8 cells were transfected with siAMPK for 72 hours and incubated in 5–30 mM D-glucose for 24 hours^4^. CQ (200 μM) or AQ (10 μM) were treated for 1 hour before changing culture medium into 30 mM D-glucose to explore the effects of the drugs on hRPTCs subjected to HG conditions. (**A**) Representative Immunoblot data showed that the effect of CQ on PGC1α phosphorylation was attenuated after inhibition of AMPK under HG condition. Band intensities representing phospho-PGC1α and PGC1α expression levels were converted into densitometry using ImageJ software in the ratio of phospho-PGC1α to PGC1α. Results are means ± SEM. for experiments in triplicate. (*p < 0.05 vs 5 mM, **p < 0.01 vs 5 mM, ^#^p < 0.05 vs 30 mM, ^##^p < 0.01 vs 30 mM, ^$$^p < 0.01 vs 30 mM + siAMPK + CQ, ^$$$^p < 0.001 vs 30 mM + siAMPK + CQ). (**B**) Inhibition of AMPK also reduced the effect of AQ on PGC1α phosphorylation under HG condition. Band intensities representing phospho-PGC1α and PGC1α expression levels were converted into densitometry using ImageJ software in the ratio of phospho-PGC1α to PGC1α. Results are means ± SEM. for experiments in triplicate. (*p < 0.05 vs 5 mM, **p < 0.01 vs 5 mM, ^#^p < 0.05 vs 30 mM, ^##^p < 0.01 vs 30 mM, ^$$^p < 0.01 vs 30 mM + siAMPK + AQ, ^$$$^p < 0.001 vs 30 mM + siAMPK + AQ).

To confirm the effect of chloroquine and amodiaquine on hRPTCs under high-glucose condition through AMPK phosphorylation, siRNA for AMPK was incubated with cells for 72 h, and then cultured in 5–30 mM D-glucose with or without treatment of chloroquine and amodiaquine. As shown in Fig. 2, the effect of chloroquine and amodiaquine on PGC1α phosphorylation in hRPTCs was attenuated after inhibition of AMPK under high-glucose condition. We observed that chloroquine and amodiaquine induce a fall in AMP/ATP ratio in hRPTCs under high-glucose environment which may be responsible for AMPK activation (Supplementary Fig. S3). We also did western blot analysis which revealed that chloroquine and amodiaquine induce LKB1 (liver kinase B1, a major upstream AMPK kinase) phosphorylation under high glucose condition (Supplementary Fig. S4).

Suppression of AMPK and PGC1α activity is linked with the pathogenesis of diabetic tubulopathy^4^. AMPK is an important regulator of cellular metabolism, and direct phosphorylation of PGC1α by AMPK increases PGC1α transcriptional activity^15^. Our findings suggest that chloroquine and amodiaquine successfully enhance AMPK phosphorylation under high-glucose conditions, resulting in increased PGC1α phosphorylation.

### Chloroquine and amodiaquine attenuate the effect of high glucose concentrations on mitochondrial biogenesis and fragmentation in RPTCs

**Figure 3:**
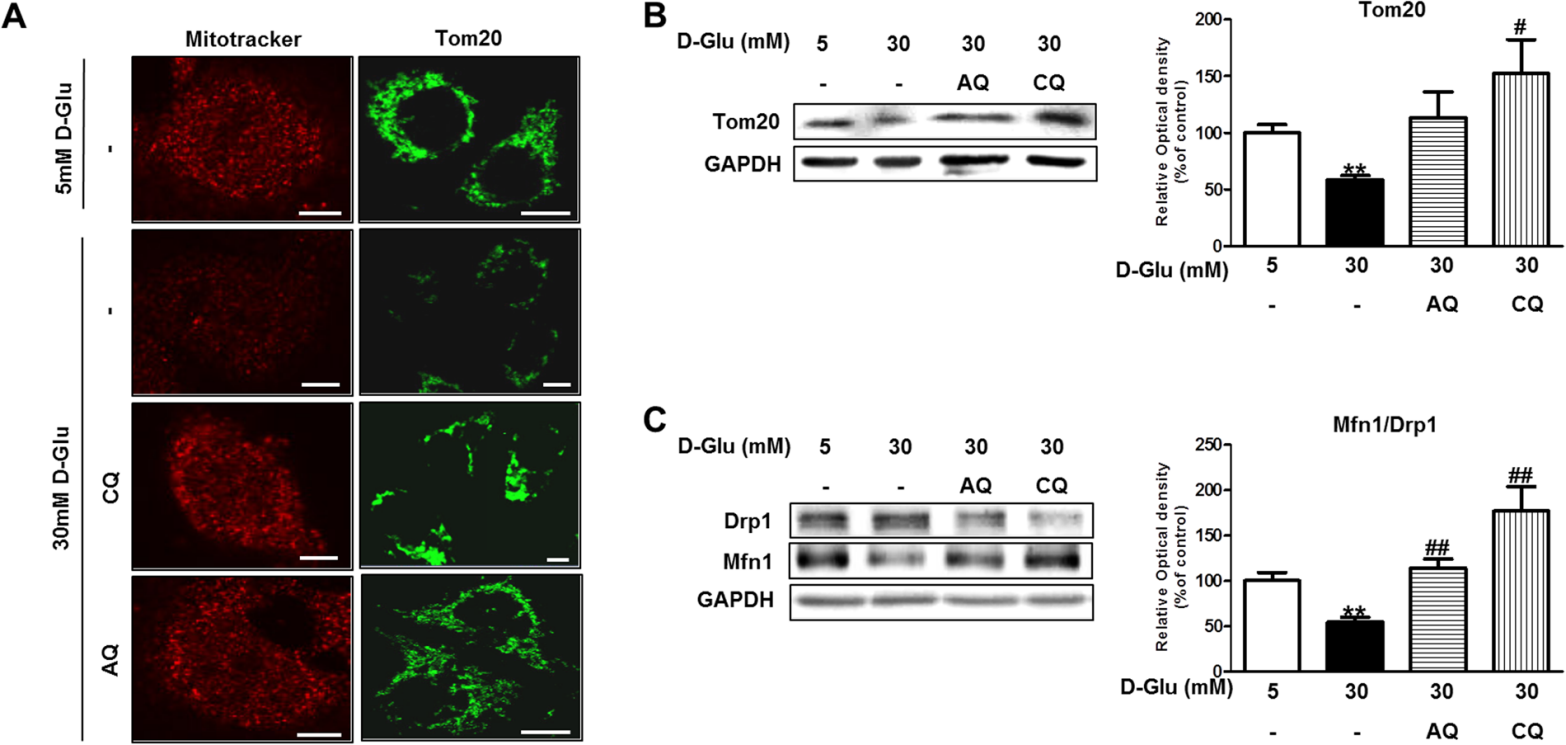
CQ and AQ ameliorated the effect of HG on mitochondrial biogenesis and fragmentation in hRPTCs. HKC-8 cells were incubated in 5–30 mM D-glucose for 24 hours and stained or harvested for analysis. CQ (200 μM) or AQ (10 μM) were treated for 1 hour before changing culture medium into 30 mM D-glucose to explore the effects of the drugs on hRPTCs subjected to HG conditions. (**A**) Cells were stained with Mitotracker (red) or anti-Tom20 antibody (green). Representative confocal fluorescence images of MitoTracker and Tom20 showing that increased numbers of functioning mitochondria were present in hRPTCs after treatment with CQ or AQ under HG conditions. (**B**) Cell lysate were immunoblotted with antibodies to Tom20 and GAPDH. Western blot analysis showed that Tom20 expression was increased in hRPTCs treated with CQ or AQ under HG conditions. Band intensities representing Tom20 and GAPDH expression levels were converted into densitometry using ImageJ software in the ratio of Tom20 to GAPDH. Results are means ± SEM. for experiments in triplicate. (**C**) Cell lysate were immunoblotted with anti-Drp1, anti-Mfn1 and anti-GAPDH antibody. Band intensities representing Mfn1 and Drp1 expression levels were converted into densitometry using ImageJ software in the ratio of Mfn1 to Drp1. A recovered Mfn1-to-Drp1 ratio was observed in hRPTCs treated with CQ or AQ and cultured under HG conditions. Results are means ± SEM. for experiments in triplicate. (**p < 0.01 vs 5 mM, ^#^p < 0.05, ^##^p < 0.01 vs 30 mM).

PGC1α is a transcriptional coactivator that regulates the genes involved in energy metabolism. PGC1α also plays important roles in mitochondrial biogenesis and function^16^. We hypothesized that chloroquine- and amodiaquine-mediated increases in PGC1α activity rescue mitochondrial abnormalities in human renal tubular cells under high-glucose conditions. MitoTracker Red stains functioning mitochondria with an intact membrane potential. Confocal microscopy showed that the number of functional mitochondria was decreased under high-glucose conditions; however, restoration of functioning mitochondrial mass was observed after treatment with chloroquine or amodiaquine (Fig. 3A). Tom20 is a component of mitochondria, and immunofluorescence and immunoblot analyses using anti-Tom20 antibodies confirmed that treatment with antimalarial drugs recovered mitochondrial mass under high-glucose conditions (Fig. 3A,B). Western blot analysis revealed that the mitochondrial profission protein Drp1 was upregulated, whereas the profusion protein Mfn1 was down-regulated in human renal tubular cells exposed to high-glucose concentrations (Fig. 3C). A low Mfn1-to-Drp1 expression ratio reflects increased mitochondrial fragmentation, which may induce cell injury^17^. Interestingly, the Mfn1-to-Drp1 expression ratio was recovered upon treatment with chloroquine or amodiaquine in high-glucose conditions (Fig. 3C).

**Figure 4:**
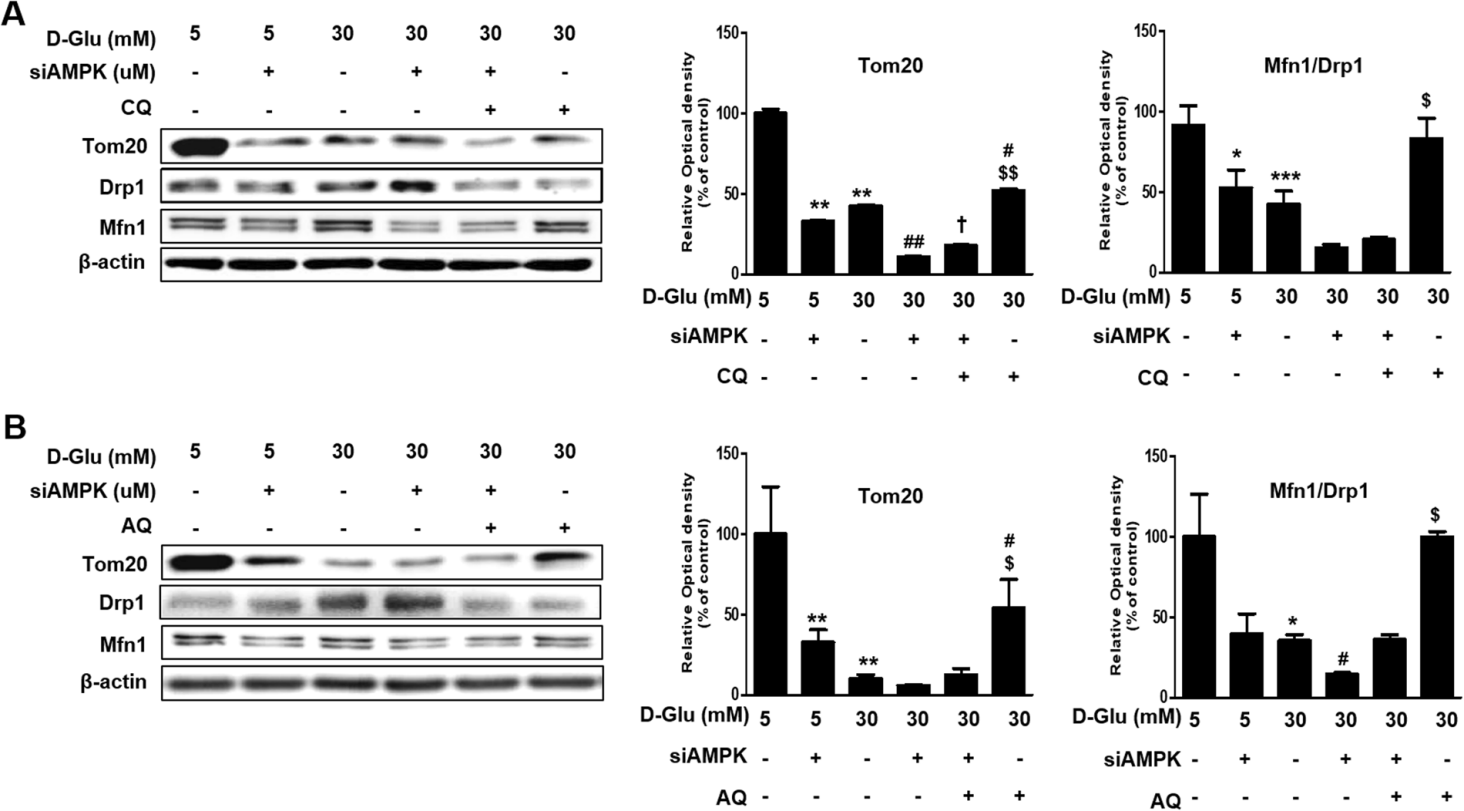
Effect of siAMPK on CQ- or AQ- induced mitochondrial biogenesis and homeostasis. (**A**) Expression levels of Tom20, Mfn1, and Drp1 in AMPK-knockdown hRPTCs under high glucose condition with or without treatment of CQ. Band intensities representing Tom20 and GAPDH expression levels were converted into densitometry using ImageJ software in the ratio of Tom20 to GAPDH. Band intensities representing Mfn1 and Drp1 expression levels were converted into densitometry using ImageJ software in the ratio of Mfn1 to Drp1. Results are means ± SEM. for experiments in triplicate. (**p < 0.01 vs 5 mM, ^#^p < 0.05, ^##^p < 0.01 vs 30 mM, ^†^p < 0.05 vs 30 mM + siAMPK, ^$^p < 0.05, ^$$^p < 0.01, ^$$$^p < 0.001 vs 30 mM + siAMPK + CQ). (**B**) Expression levels of Tom20, Mfn1, and Drp1 in AMPK-knockdown hRPTCs under high glucose condition with or without treatment of AQ. Band intensities representing Tom20 and GAPDH expression levels were converted into densitometry using ImageJ software in the ratio of Tom20 to GAPDH. Band intensities representing Mfn1 and Drp1 expression levels were converted into densitometry using ImageJ software in the ratio of Mfn1 to Drp1. Results are means ± SEM. for experiments in triplicate. (**p < 0.01 vs 5 mM, ^#^p < 0.05, ^##^p < 0.01 vs 30 mM, ^$^p < 0.05, ^$$^p < 0.01 vs 30 mM + siAMPK + AQ).

We examined the effects of chloroquine and amodiaquine on mitochondrial dynamics after inhibition of AMPK. The siRNA for AMPK significantly inhibited chloroquine- or amodiaquine-mediated enhancement of TOM20 expression in hRPTCs subject to high glucose environment (Fig. 4A,B). Also, AMPK knocked down significantly attenuated normalization of Mfn1-to-Drp1 ratio by chloroquine and amodiaquine in hRPTCs under high glucose condition (Fig. 4A,B). Thus, these results suggest that chloroquine and amodiaquine improve mitochondrial dynamics probably through AMPK activation under high glucose conditions.

### Chloroquine and amodiaquine reduce mitochondrial ROS production and apoptosis in RPTCs in a high-glucose environment

**Figure 5:**
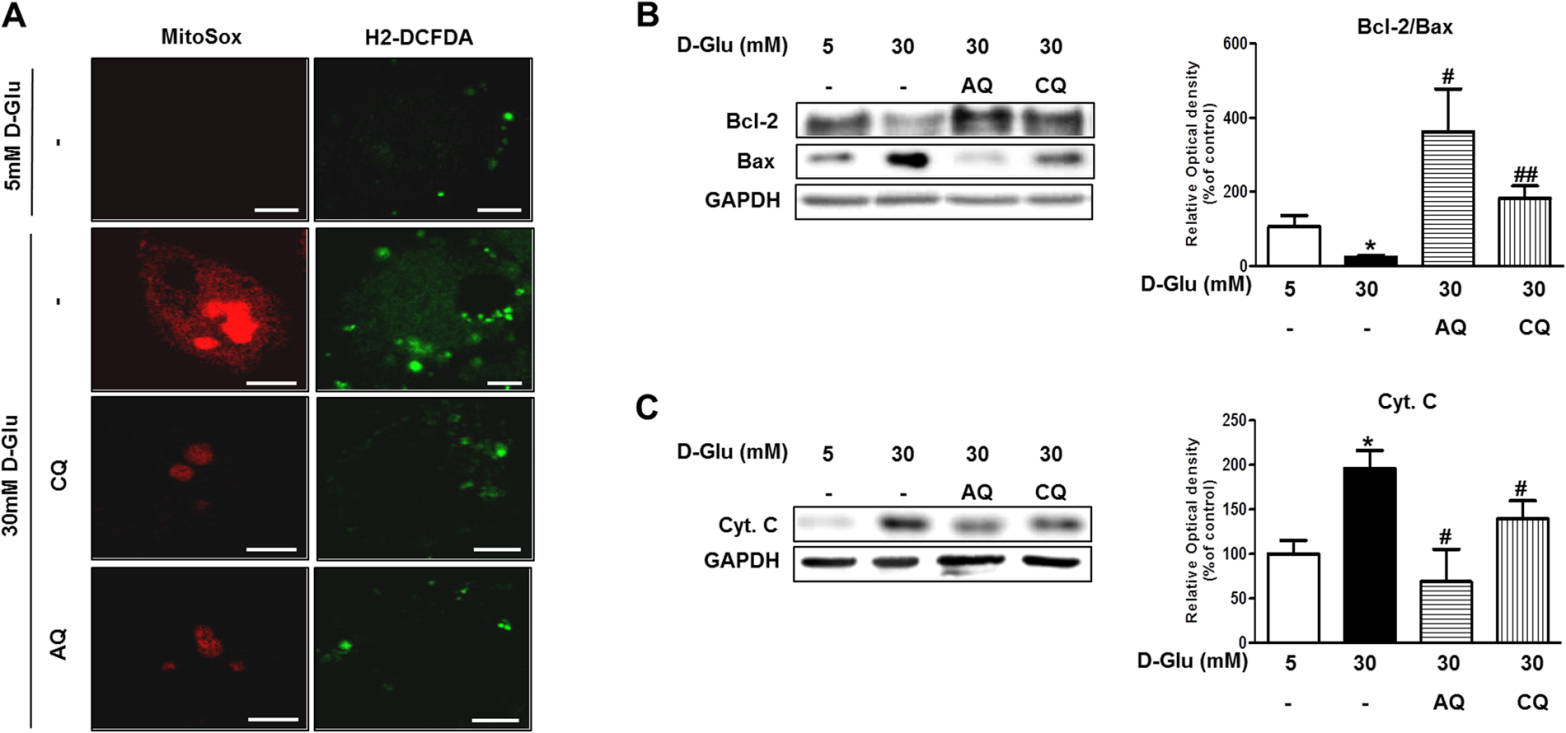
Effect of CQ and AQ on ROS production and apoptosis in hRPTCs under HG conditions. HKC-8 cells were incubated in 5 or 30 mM D-glucose for 24 hours and used for analysis. CQ (200 μM) or AQ (10 μM) were pretreated for 1 hour before exposure to 30 mM D-glucose for investigating effect of the drugs on hRPTCs under HG conditions. (**A**) Representative confocal images of MitoSox (red) and H2-DCFDA (green) showing that mitochondrial and intracellular ROS production was reduced in CQ- and AQ-treated hRPTCs under HG conditions. (**B**,**C**) Western blot analyses revealed that the expression of the apoptogenic proteins Cyt. C and Bax was decreased, while the expression of the anti-apoptotic protein Bcl2 was increased after CQ or AQ treatment under HG conditions. Band intensities representing Bcl-2, Bax, Cyt. C and GAPDH expression levels were converted into densitometry using ImageJ software in the ratio of Bcl2 to Bax and Cyt. C to GAPDH. Results are means ± SEM. for experiments in triplicate. (*p < 0.05 vs 5 mM, ^#^p < 0.05, ^##^p < 0.01 vs 30 mM, Scale bar 10 μm).

We subsequently investigated mitochondrial ROS production and apoptosis-related protein expression to determine the effect of the recovery of mitochondrial abnormalities in HKC8 cells under high-glucose conditions. Higher-intensity staining with MitoSOX and H2-DCFDA probes suggested that high glucose concentrations induced mitochondrial and cytosolic ROS generation in HKC8 cells (Fig. 5A). However, treatment with chloroquine or amodiaquine significantly reduced mitochondrial and cytosolic ROS production, changes reflected by reductions in MitoSOX and H2-DCFDA probe intensity in HKC8 cells under high-glucose conditions (Fig. 5A).

Immunoblot analysis revealed that the above decreases in ROS production were accompanied by decreased expression of the apoptogenic Bax and Cytochrome C (Cyt. C) in RPTCs treated with chloroquine or amodiaquine compared with cells treated without chloroquine or amodiaquine under high-glucose conditions (Fig. 5B,C). In addition, chloroquine and amodiaquine increased the expression of the antiapoptotic Bcl-2, resulting in a low Bcl-2-to-Bax expression ratio in RPTCs cultured under high-glucose conditions.

Taken together, the above findings show that treatment with chloroquine and amodiaquine reduced mitochondrial ROS production and created an environment favorable for the survival of RPTCs by regulating apoptosis-related proteins even under high-glucose conditions.

### Chloroquine and amodiaquine diminish transforming growth factor (TGF)-β1 expression and attenuate epithelial-to-mesenchymal transition (EMT) in RPTCs under high-glucose conditions

TGF-β1 is a central cytokine in renal fibrosis and has multiple functions in renal inflammation and apoptosis^18^. Dysregulated TGF-β1 expression and activation stimulate extracellular matrix production, leading to the development of tubulointerstitial fibrosis^18^. We assessed the effects of chloroquine and amodiaquine on the expression of TGF-β1 and associated tubular EMT markers under high-glucose conditions.

**Figure 6:**
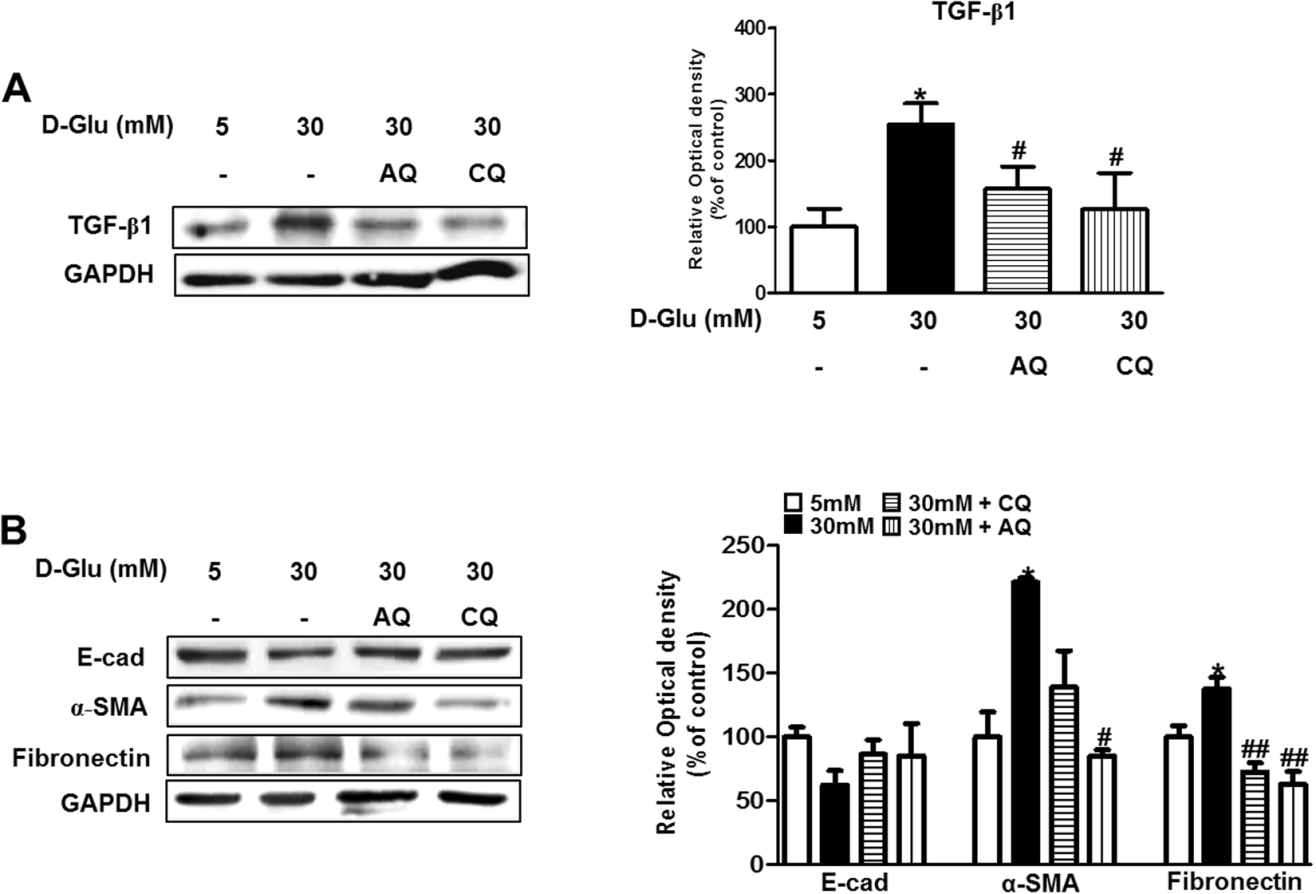
CQ and AQ reduced TGF-β1 expression and the EMT phenotype in hRPTCs under HG conditions. HKC-8 cells were incubated in 5–30 mM D-glucose for 24 hours and collected for analysis. CQ (200 μM) or AQ (10 μM) were treated for 1 hour before changing culture medium into 30 mM D-glucose to figure out how the drugs effect on hRPTCs under HG conditions. (**A**) Immunoblotting showed that TGF-β1 expression was decreased after treatment with CQ or AQ under HG conditions. Band intensities representing TGF-β1 and GAPDH expression levels were converted into densitometry using ImageJ software in the ratio of TGF-β1 to GAPDH. Results are means ± SEM. for experiments in triplicate. (**B**) Western blot analysis revealed that the expression of the epithelial marker E-cadherin (E-Cad) was restored, whereas that of the mesenchymal markers α-SMA and fibronectin was decreased after CQ or AQ treatment under HG conditions. Band intensities representing E-cad, α-SMA, fibronectin and GAPDH expression levels were converted into densitometry using ImageJ software in the ratios of E-cad, α-SMA, and fibronectin to GAPDH. Results are means ± SEM. for experiments in triplicate. (*p < 0.05 vs 5 mM, ^#^p < 0.05, ^##^p < 0.01 vs 30 mM).

As shown by Fig. 6A, Chloroquine and amodiaquine suppressed TGF-β1 expression, which was upregulated in high-glucose conditions, and attenuated the increases in 𝛼-smooth muscle actin (SMA) and fibronectin expression induced by high glucose concentrations. However, the expression of E-cadherin, which is a marker of the epithelial phenotype, was non-significantly increased in cells incubated with chloroquine or amodiaquine under high-glucose conditions (Fig. 6B).

The roles of tubular EMT in the progression of kidney fibrosis remain controversial^19–21^. However, partial EMT, the process by which mesenchymal marker expression increases in renal epithelial cells without the loss of cell-to-cell adhesion and polarity, has been proposed as the link between acute or continuous renal injury and kidney fibrosis^19^. As illustrated by our results, chloroquine or amodiaquine abolished high-glucose-induced dysregulation of TGF-β1 expression and suppressed partial EMT in RPTCs under high-glucose conditions.

### Ameliorative effects of chloroquine and amodiaquine on albuminuria and renal histology, as reflected by enhancements of AMPKα phosphorylation, in STZ-induced diabetes in mice

To determine whether chloroquine and amodiaquine are effective against diabetic kidney disease *in vivo*, we injected chloroquine or amodiaquine into STZ-induced diabetic mice for a period of 14 weeks.

**Figure 7:**
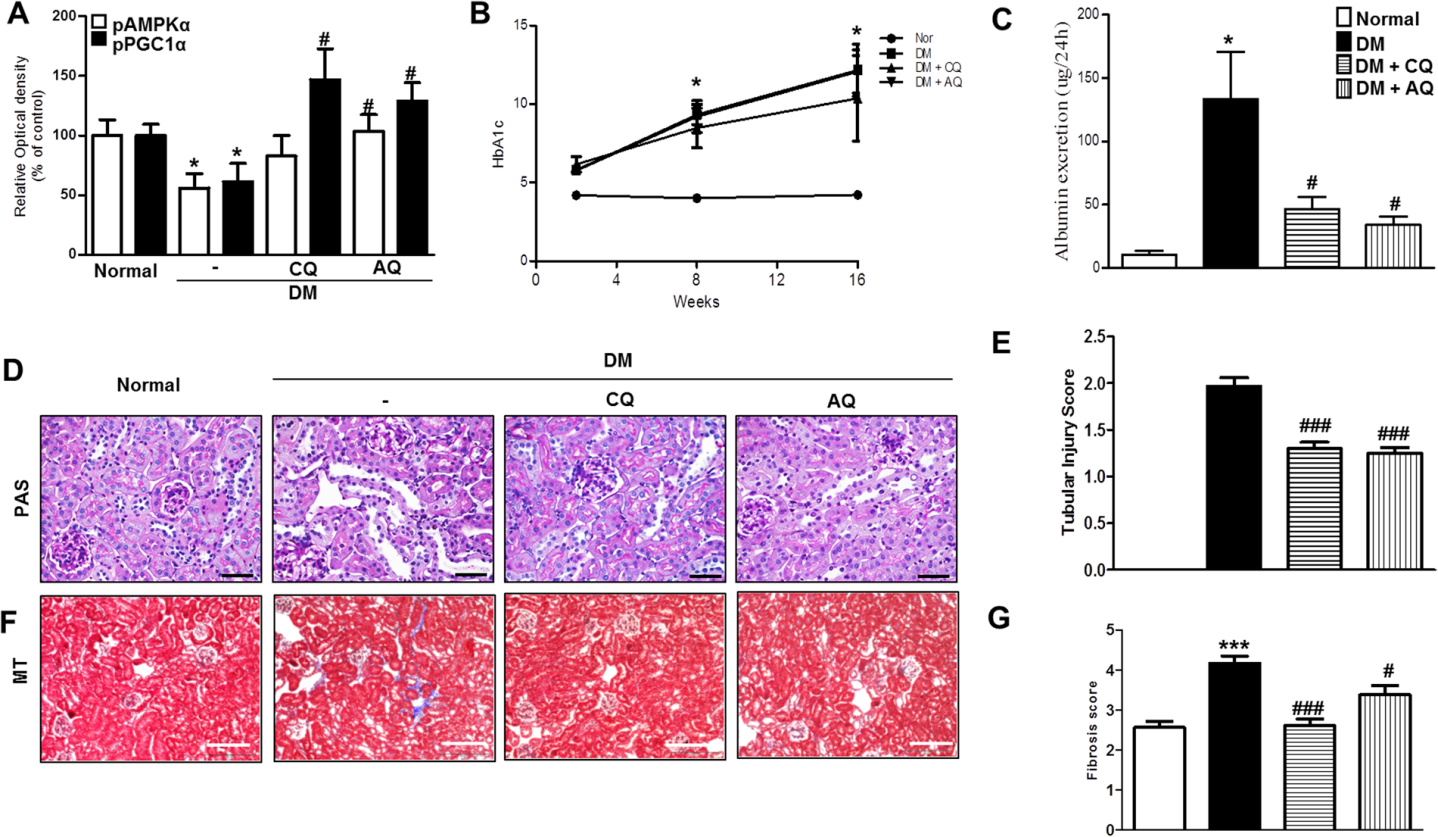
CQ and AQ increased pAMPKα expression and ameliorated albuminuria and renal morphologic changes in the kidneys of STZ-induced diabetic mice. CQ (50 mg/kg) or AQ (20 mg/kg) were administered every other day for 14 weeks in treatment groups. (**A**) pAMPKα and pPGC1α activity restoration was confirmed by immunoblot analysis in diabetic kidneys treated with CQ or AQ. Band intensities representing pAMPKα, pPGC1α and GAPDH expression levels were converted into densitometry using ImageJ software in the ratios of E-cad, α-SMA, and fibronectin to GAPDH. Data are means ± SEM. (**B**) The STZ-induced diabetes group had higher HgA1C levels than the normal control group. (**C**) However, CQ- and AQ-treated STZ-induced diabetic mice exhibited a significant reduction in the urine albumin excretion rate compared with diabetic control mice. (**D**,**E**) Tubular dilatation and tubular epithelial disruption were observed in the diabetic control group. Treating STZ-induced diabetic mice with CQ or AQ attenuated these abnormalities while causing less cellular disruption. (**F**,**G**) Representative of photographs of Masson’s trichrome-stained kidneys showing that decreased numbers of renal fibrotic lesions were present in the CQ and AQ groups compared with the diabetic control group. (n = 5 per group, *p < 0.05, ***p < 0.0001 vs Normal, ^#^p < 0.05, ^###^p < 0.0001 vs DM control).

Immunoblot analysis confirmed that AMPKα and PGC1α activity was restored in the kidneys of diabetic mice treated with chloroquine or amodiaquine (Fig. 7A, and see Supplementary Fig. S5). STZ-induced diabetic mice had higher HbA1C levels than normal mice, regardless of whether they received chloroquine or amodiaquine during the study (Fig. 7B). However, chloroquine and amodiaquine treatment induced significant reductions in urine albumin excretion in diabetic mice but had no effect on blood glucose levels (Fig. 7C).

Morphology studies using Periodic acid–Schiff (PAS) staining revealed that different types of tubular injury, such as tubular epithelial disruption and tubular dilatation, occurred in diabetic mouse kidneys. However, the kidneys of diabetic mice treated with chloroquine or amodiaquine displayed improvements in the tubular compartment while exhibiting less cellular disruption compared with those in the diabetic control group (Fig. 7D). Semiquantitative analysis based on damage scores showed that both chloroquine and amodiaquine alleviated tubular damage (Fig. 7E). Masson’s trichrome staining showed that the number of renal fibrotic lesions was decreased in the chloroquine and amodiaquine groups compared with the diabetic control group (Fig. 7F). Semiquantitative determination revealed that fibrosis scores were significantly lower in the kidneys of diabetic mice treated with chloroquine or amodiaquine than in those of diabetic control mice (Fig. 7G).

### Chloroquine and amodiaquine restore mitochondrial mass, attenuate fragmentation and contribute to decreases in oxidative stress in the renal tubules of STZ-induced diabetic mice

**Figure 8:**
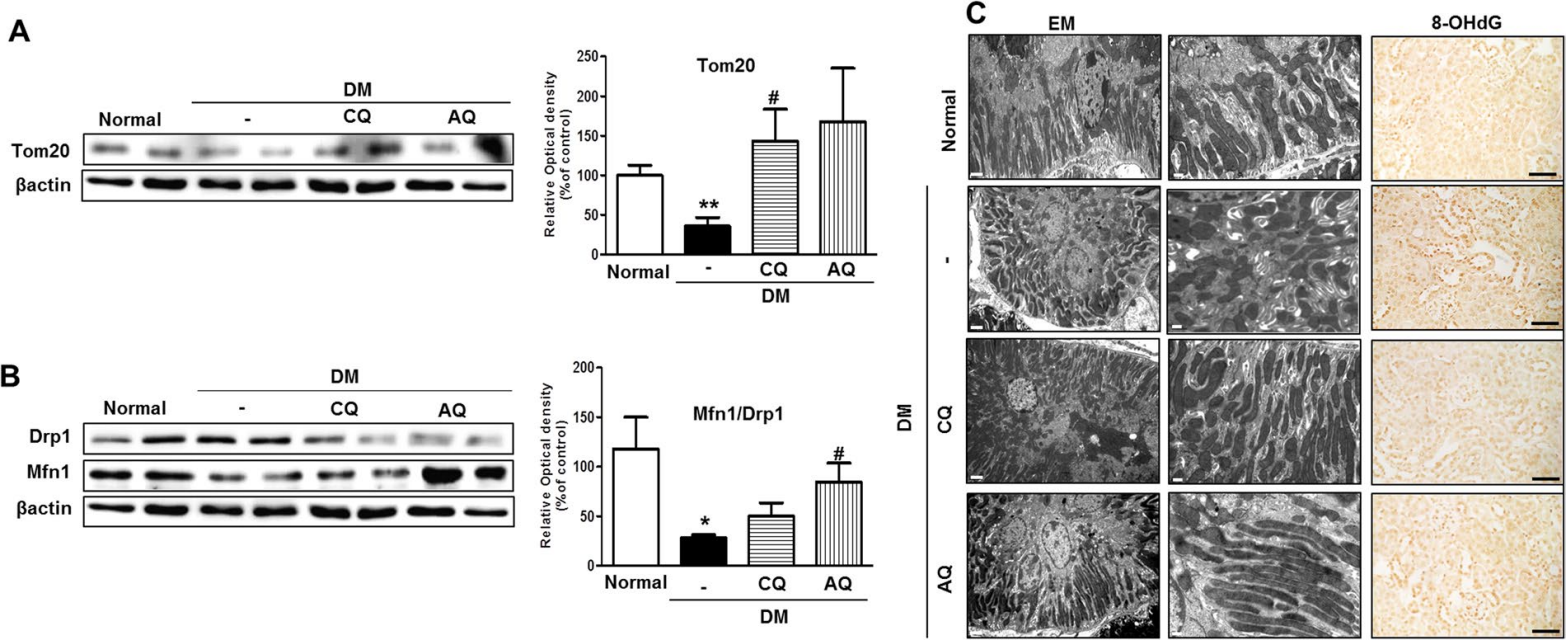
CQ and AQ restored mitochondrial abnormalities and reduced oxidative stress in STZ-induced diabetic mice. (**A**) Immunoblotting showed that Tom20 expression was increased in diabetic kidneys after treatment with CQ or AQ. Band intensities representing Tom20 and βactin expression levels were converted into densitometry using ImageJ software in the ratios of Tom20 to βactin. Data are means ± SEM. (n = 5 per group, **p < 0.01 vs Normal, ^#^p < 0.05 vs DM control). (**B**) Western blot analysis revealed that CQ and AQ suppressed Drp1 expression but increased Mfn1 expression, resulting the normalization of the Mfn1-to-Drp1 ratio in diabetic kidneys. Band intensities representing Mfn1 and Drp1 expression levels were converted into densitometry using ImageJ software in the ratios of Mfn1 to Drp1. Data are means ± SEM. (n = 5 per group, *p < 0.05 vs Normal, ^#^p < 0.05 vs DM control). (**C**) Representative images of electron micrographs showing elongated mitochondria in the renal tubular cells of the normal control group. Most of the mitochondria were short or spherical in shape in the diabetic group. However, CQ or AQ administration markedly attenuated mitochondrial fragmentation in the renal tubular cells of diabetic kidneys. (Scale bar 2 and 0.5 μm). (**D**) Immunoperoxidase staining showing that tubular 8-OHdG content was increased in diabetic kidneys but was effectively reduced by treatment with CQ or AQ. (Scale bar 100 μm).

We explored the effects of chloroquine and amodiaquine on functional mitochondrial mass and mitochondrial fission in diabetic kidneys. Western blot analysis revealed that the expression of the mitochondrial import receptor subunit Tom20 was increased in the diabetic kidneys of the mice in the chloroquine and amodiaquine groups compared with those in the diabetic control group (Fig. 8A). Figure 8B shows that chloroquine and amodiaquine successfully restored Drp1 and Mfn1 expression, resulting in the normalization of Mfn1-to-Drp1 expression ratio in diabetic kidneys. Electron microscopic analysis revealed that the renal tubular cells of normal mice contained numerous elongated mitochondria organized along membrane compartments. In contrast, the mitochondria in the renal tubular cells of diabetic control mice were small and disorganized (Fig. 8C). However, treatment with chloroquine or amodiaquine markedly attenuated mitochondrial fission in the renal tubular cells of chloroquine- and amodiaquine-treated mice (Fig. 8A). Increase of 8-oxo-2′-deoxyguanosine (8-OHdG) in cells is a measurement of oxidative stress. Peroxidase staining revealed that tubular 8-OHdG content was decreased in the kidneys of the chloroquine and amodiaquine groups but was increased in the renal tubules of the diabetic control group (Fig. 8D).

### Chloroquine and amodiaquine improve the renal expression of apoptogenic and EMT-related proteins in the kidneys of STZ-induced diabetic mice

**Figure 9:**
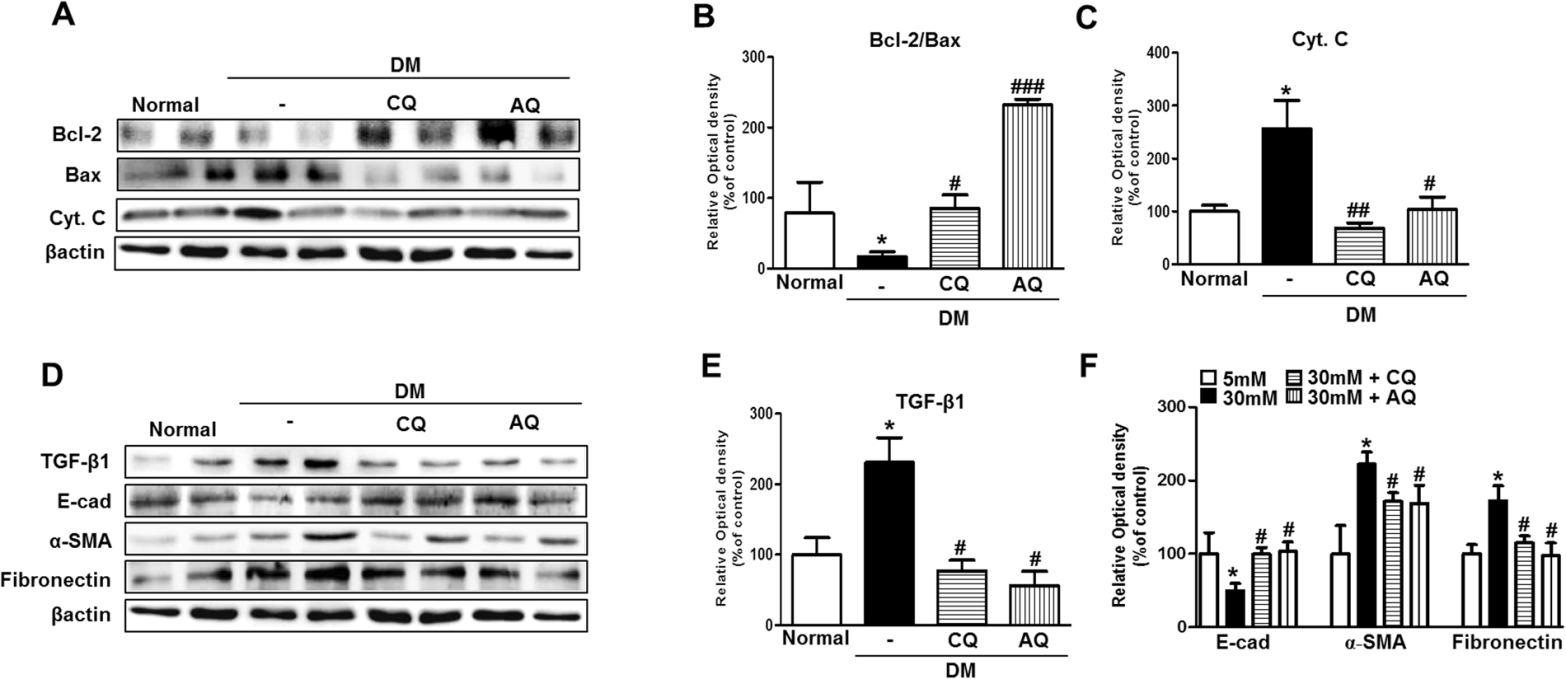
CQ and AQ treatment attenuated the renal expression of apoptogenic and EMT-related proteins in diabetic kidneys. (**A**–**C**) The expression of the apoptogenic proteins Bax and Cyt. C was markedly increased, while that of the anti-apoptogenic Bcl-2 was reduced in diabetic kidneys. However, CQ or AQ treatment ameliorated the changes in the expression of the apoptosis- and anti-apoptosis-related proteins. Band intensities representing Bcl-2, Bax, Cyt.C and βactin expression levels were converted into densitometry using ImageJ software in the ratios of Bcl-2 to Bax and Tom20 to βactin. Data are means ± SEM (n = 5 per group, *p < 0.05 vs Normal, ^#^p < 0.05, ^##^p < 0.01, ^###^p < 0.0001 vs DM control). (**D**–**F**) Increased TGF-ß1, α-SMA and fibronectin expression was observed in the kidneys of the diabetic group, whereas diminished TGF-ß1, α-SMA and fibronectin expression was observed in the CQ and AQ groups. The expression of E-cad, the epithelial cell marker protein, was recovered by treatment with CQ and AQ in diabetic kidneys. Band intensities representing TGF-β1, E-cad, α-SMA, fibronectin and βactin expression levels were converted into densitometry using ImageJ software in the ratios of TGF-β1, E-cad, α-SMA, and fibronectin to βactin. Data are means ± SEM (n = 5 per group, *p < 0.05 vs Normal, ^#^p < 0.05 vs DM control).

We expected that drug-induced improvements in mitochondrial abnormalities and reductions in oxidative stress will provide conditions favorable for the survival of renal tubular cells under diabetic conditions^4^. We investigated the renal expression of apoptogenic proteins in the kidneys of normal control mice, diabetic control mice and diabetic mice treated with chloroquine or amodiaquine. Notably, western blot analysis revealed that the expression of the anti-apoptotic protein Bcl-2 was increased, and the expression of the apoptogenic proteins Bax and Cyt. C was decreased in the chloroquine and amodiaquine treatment groups compared with the diabetic control group (Fig. 9A,C). Figure 9B showed treatment with chloroquine or amodiaquine were associated with high ratio of Bcl2-to-Bax expression ratio in diabetic kidneys. Furthermore, treatment with chloroquine and amodiaquine greatly reduced TGF-β1 expression in diabetic kidneys (Figs 9D and 8E), a change accompanied by the restoration of the epithelial marker E-cadherin and the suppression of α-SMA and fibronectin (Fig. 9D,F).

## Discussion

Diabetes is the typical pathological consequence of an imbalance of energy homeostasis^15^. Mitochondria are the tiny intracellular organelles responsible for energy production^22–24^, and a growing body of evidence indicates that defects in mitochondrial function and dynamics are crucial for the development of diabetes and its complications^25–27^. Here, we showed that chloroquine and amodiaquine induce mitochondrial biogenesis and balance fusion/fission protein expression in RPTCs under high-glucose conditions and in diabetic kidneys, presumably by enhancing AMPK and PGC1α phosphorylation and activity. Both chloroquine and amodiaquine alleviate diabetic tubulopathy, a change accompanied by decreases in albuminuria and improvements in renal tubulointerstitial pathology in STZ-induced diabetic mice.

Recent studies showed that dynamic morphological changes occur in mitochondria during cell injury or stress and that these changes contribute to mitochondrial damage and, consequently, cell death^28^. Mitochondrial fission causes the production of short mitochondrial spheres, while fusion promotes the production of long, filamentous mitochondria^28^. Under physiologic conditions, both fission and fusion occur continuously and are finely controlled by associated modulators^29–35^. Drp1, a member of the dynamin family of large GTPases, play a key role in mitochondrial fission, as it facilitates the formation of spirals to drive mitochondrial outer membrane (MOM) cleavage^36^. Other proteins, such as Fis1, Bif-1 and endophilin B1, are also reported to be components of the mitochondrial fission machinery^37–39^. Mfn1, Mfn2 and optic atrophy 1 (OPA1) are the basic components of the mitochondrial fusion machinery^40^. Mfn1/2 mediate the fusion of MOM with adjacent mitochondria, and OPA1 is responsible for the union of the mitochondrial inner membrane^40^. Notably, increased numbers of fragmented mitochondria have been observed in renal diseases, such as diabetic nephropathy^4,17,41^. Importantly, excessive fragmentation has been demonstrated to have pathologic effects during the development and progression of the disease^28^. The consistent results of a previous study suggest that mitochondrial fission triggers mitochondrial outer membrane permeabilization (MOMP), Bax activation, Cyt. C leakage and cell death^28^.

We previously reported that mitochondrial abnormalities developed in RPTCs exposed to a high-glucose environment and demonstrated that PGC1α upregulation compensated for the deleterious effects of high glucose on mitochondrial biogenesis and dynamics in the RPTCs and kidneys of STZ-induced diabetic mice^4^. At this time, we observed that chloroquine and amodiaquine enhanced AMPK phosphorylation, which led to increased PGC1α activity RPTCs under high-glucose conditions (Figs 1–2). PGC1α is a master regulator of mitochondrial biogenesis, and its activity can be tuned in response to different metabolic situations^15^. PGC1α functions as an energy regulator by directly coactivating multiple transcription factors, including PPARα, myocyte enhancer factor-2, estrogen-related receptors, and nuclear respiratory factor-1/2, which govern mitochondrial DNA transcription, replication, or function^15,42,43^. AMPK is the important gate keeper that activates PGCα by phosphorylation^15^. Our data showed that chloroquine- and amodiaquine-induced increases in PGC1α activity overcome the effects of high glucose on mitochondrial mass and fragmentation and ROS generation in hRPTCs (Figs 3, 5). These effects of chloroquine and amodiaquine on PGC1α activation and mitochondrial homeostasis in hRPTCs under high-glucose conditions were attenuated after the inhibition of AMPK using siRNA, suggesting the drugs action in diabetic tubulopathy presumably through AMPK. However, it is one of the limitation of our results in that siRNA has disadvantages, such as the variability and incompleteness of knockdowns and the potential nonspecificity of reagents.

Chloroquine and amodiaquine also reduce the expression of apoptotic (Bax and Cyt. C) and fibrotic proteins (TGF-β1, α-SMA, and fibronectin) but increase the expression of an anti-apoptotic protein (Bcl-2) by correcting mitochondrial abnormalities in renal proximal tubular epithelial cells, even under high-glucose conditions (Figs 5, 6). Similarly, both chloroquine and amodiaquine attenuated disruptions of mitochondrial homeostasis in the kidneys of STZ-induced diabetic mice (Fig. 8). STZ-induced diabetic mice exhibited concurrent reductions in albuminuria and improvements in renal tubular injury and tubulointerstitial fibrosis (Fig. 7D–G). Examination of the tubular cells of diabetic mice via electron microscopy showed that most of the mitochondria were short and fragmented; however, after chloroquine or amodiaquine treatment, the mitochondria became longer and more filamentous, changes indicative of fusion (Fig. 8C). Figure 8D shows that more severe oxidative stress in the kidney was tied to excessive mitochondrial fragmentation in the renal tubules. We confirmed that the expression of apoptotic proteins was decreased and that the expression of EMT markers and TGF-β1 was lower in the kidneys of diabetic mice in the chloroquine and amodiaquine treatment groups compared with those in the kidneys of mice in the diabetic control group (Fig. 9).

AMPK is a heterotrimeric complex comprised of a catalytic α and regulatory β and γ subunits. AMPK can be activated by a decrease in ATP accompanying with an increase in ADP and AMP. As AMP binds to the γ subunit, AMPK undergoes a conformational change which facilitates the α subunit being a substrate for phosphorylation at Thr172 by LKB1 (liver kinase B1, a major upstream AMPK kinase). Calcium-calmodulin-dependent kinase kinase 2 (CaMKK2), and TGFβ-activated kinase 1 (TAK1) are also involved in the phosphorylation of AMPK at Thr172, while protein phosphatase 2A (PP2A), protein phosphatase 2C (PP2C) and Mg2+−/Mn2+-dependent protein phosphatase 1E (PPM1E) dephosphorylate AMPK causing inactivation^44^. We observed that chloroquine and amodiaquine induce a fall in AMP/ATP in hRPTCs under high-glucose conditions, which may be responsible for the AMPK activation. Chloroquine and amodiaquine also contribute to phosphorylate LKB1 in hRPTCs subject to high glucose. However, other potential participants in the mechanism, including CaMKK2, TAK1, PP2C and PPM1E, were not investigated in our study.

Diabetic glomerulopathy used to be considered the major feature of DKD; however, more recent findings suggest that diabetic tubulopathy plays a leading role in the development and progression of DKD and is thus not merely a bystander in or consequence of the development of this disease^45,46^. Hasegawa *et al*. provided evidence showing that SIRT1 expression is abnormally depressed in renal proximal tubule-induced glomerular podocytopathy in diabetic mice^46^. Moreover, Grgic *et al*. demonstrated that targeted renal tubular injury was sufficient to cause glomerulosclerosis, interstitial fibrosis, and albuminuria in mice whose renal epithelial cells expressed the diphtheria toxin receptor^47^. A recent study showed that mitochondrial functional impairments and organellar fragmentation occurred earlier than albuminuria in the renal proximal tubular epithelial cells of diabetic mice, suggesting that mitochondrial abnormalities are an appropriate target for the prevention of diabetic tubulopathy^48^.

Unfortunately, we did not investigate whether the drugs are having glomerular effects as well as tubular effects. In our opinion, both indirect effects via renal tubules and direct effects of chloroquine and amodiaquine on renal glomerulus are possible. Indeed, Eid *et al*. reported that the activation of AMPK prevents glomerular epithelial cell apoptosis in diabetic mice^49^. Additional studies are needed to investigate the roles of chloroquine and amodiaquine in glomerular cells in diabetes.

Chloroquine has been used safely as a long-term maintenance drug in patients with lupus alone or combined lupus glomerulonephritis. In our study, chloroquine and its derivative, amodiaquine, effectively ameliorated diabetic tubulopathy *in vivo* and *in vitro*. Chloroquine and amodiaquine play roles in the restoration of mitochondrial biogenesis and the attenuation of unbalanced fragmentation by facilitating AMPK phosphorylation following increases in PGC1α activation. Our results suggest that chloroquine and amodiaquine may be useful as treatments for patients with DKD.

## Materials and Methods

### Cell Culture

The human renal proximal tubular epithelial cell line HKC-8 was obtained from Dr. L. Rausen (Johns Hopkins University, Baltimore, MD) and was maintained in Dulbecco’s modified Eagle medium supplemented with Ham’s F12 medium (DMEM/F12; Invitrogen, CA, USA). DMEM/F12 was supplemented with 10% fetal bovine serum and 1% penicillin/streptomycin (WelGENE, Daegu, and Republic of Korea). For the experiments, HKC8 cells were cultured with 5–30 mM D-glucose for 6–24 hours^4^ and then cells were collected for analysis. To explore the effects of the drugs on hRPTCs subjected to high-glucose condition, chloroquine (200 μM; Sigma-Aldrich St. Louis, MO, USA) or amodiaquine (10 μM; Sigma-Aldrich, St. Louis, MO, USA) were treated for 1 hour before changing culture medium into 30 mM D-glucose (4). Each cellular experiments have been performed in triplicate.

### Ethics statement

This study was carried out in strict accordance with the recommendations in the Guide of the Care and Use of Laboratory Animals of the Korea National Institutes of Health (KNIH). All animal experiments were performed in compliance with the guidelines of the Animal Research Ethics Committee of Kyung Hee University and Institutional Animal Care and Use committee Kyung Hee University Hospital at Gangdong, Seoul, Korea (approval number: KHNMC AP 2017-010).

### Animal Studies

Diabetes was induced in eight-week-old male C57/BL6J mice (Center for Research Animals, Seoul, Korea) by the intraperitoneal injection of STZ (Sigma Chemicals, Missouri) at a dose of 50 mg/kg for 5 consecutive days. In the intervention study, the following four groups of mice (*n* = 5 in each group) were used for three separate experiments: (1) a normal control group, (2) a diabetic control group, (3) a diabetes + chloroquine (50 mg/kg) group, and (4) a diabetes + amodiaquine (20 mg/kg) group. Chloroquine (Sigma-Aldrich, St. Louis, MO, USA) and amodiaquine (Sigma-Aldrich, St. Louis, MO, USA) were dissolved in saline and administered to the mice at the indicated doses via intraperitoneal injections at 48-h intervals for 14 weeks beginning 2 weeks after STZ administration. All the mice were sacrificed 16 weeks after STZ administration, and their kidney tissues were collected for analysis. During the experiments, bodyweights and serum glucose concentrations were measured weekly.

### Western Blot Analysis

Cells and kidney tissues were washed with PBS and lysed in ice-cold lysis buffer containing a protease inhibitor cocktail (Roche Diagnostics, Mannheim, Germany). The proteins were separated with 8–15% SDS-PAGE and then transferred onto a polyvinylidene difluoride membrane (Millipore, Madrid, Spain) by electroblotting. The experiments were performed according to the manufacturer’s protocols. Briefly, the membrane was blocked for 1 h at room temperature and then incubated with the following primary antibodies overnight at 4 °C: anti-PGC-1α, anti-Tom20, anti-Bax, anti-Cytochrome C, anti-E-cadherin, anti-Fibronectin (1:1000, Santa Cruz, USA), anti-phospho-PGC-1α (R&D Systems Inc. Minneapolis, MN), anti-AMPK, anti-phospho-AMPK, anti-TGF-β, anti-Bcl-2 (1:1000, Cell Signaling Technology, MA, USA), anti-Drp1, anti-Mfn-1, and α-SMA (1:1000 Abcam Inc. Cambridge, UK). The membranes were subsequently stained with horseradish peroxidase-conjugated goat anti-rabbit or mouse immunoglobulin G (1:2,000, Santa Cruz CA, USA). The immunoreactive bands were detected by chemiluminescence (Enhanced Chemiluminescence; BioFX Laboratories, Inc., Maryland). GAPDH (1:2,000, Santa Cruz, CA, USA) or β-actin (1:2,000, Santa Cruz, CA, USA) was used as an internal control.

### Transfection of siRNA into cells

Transient transfection of siRNA was carried out according to Santa Cruz’s protocol. The siRNAs were dissolved in siRNA buffer (20 mM KCl; 6 mM HEPES, pH 7.5; 0.2 mM MgCl2) to prepare a 10 mM stock solution. Cells grown in 6-well plates were transfected with siRNA in transfection medium (DMEM/F12 with free 10% fetal bovine serum and 1% penicillin/streptomycin) containing liposomal transfection reagent (Lipofectamine 2000, Invitrogen). For each transfection, 200 μl transfection medium containing 10 μl siRNA stock solution was gently mixed with 200 μl transfection medium containing 10 μl transfection reagent. After 15~30 min incubation at room temperature, siRNA-lipid complexes were added to the cells in 2 ml transfection medium, and cells were incubated with this mixture in the absence of serum for 48~72 hours at 37 °C. The cells at 40–60% confluence were transfected with siRNAs. After the incubation with siRNA (100 nM) solution for 48 h, cells were treated with chloroquine 200 μM or amodiaquine 10 μM for 1 h, and then incubated with DMEM/F12 under 5–30 mM D-glucose conditions for an additional 24 h.

### Determination of cellular AMP/ATP ratio

ATP/AMP ratio was determined using a Cellular Adenine Nucleotides-Glo™ assay kit (CAS2031A, Promega) with modifications. Cells were seeded at 10,000 cells/well in 96-well plate. After 24 hours, the cells were treated with chloroquine (200 μM) and amodiaquine (10 μM) for 1 hour and then cultured in 5–30 mM D-glucose for 24 hours. The cells were determined using a cell based bioluminescent assay system; the reagents produce a light signal from ATP, ADP and AMP by using luciferase/luciferin reaction from cells directly. Data are explained to Luminescent content (% of 5 mM D-glucose group)

### Measurement of Reactive Oxygen Species (ROS)

To assess intracellular and mitochondrial ROS production, we incubated the cells with H2-DCFDA and MitoSOX (Life Technologies, Seoul, and Republic of Korea) according to the manufacturer’s instructions, after which we examined them by confocal microscopy (LSM-700; Carl Zeiss, Jena, Germany).

### Immunofluorescence

Cells were fixed with paraformaldehyde, permeabilized, blocked with bovine serum albumin (BSA), and then incubated with the appropriate primary antibodies. After being washed with PBS, the cells were re-incubated with secondary antibodies conjugated with Alexa Fluor 488 or 594 (Life Technologies, Korea). For kidney tissue immunofluorescence, we prepared 4-μm-thick cryostat sections, mounted them on glass slides, air-dried them, and then rehydrated them with PBS. Similarly, 3-μm-thick paraffin-embedded sections were prepared, dewaxed, and re-hydrated. After being blocked with BSA, the sections were incubated with the appropriate primary antibodies, followed by Alexa Fluor-conjugated secondary antibodies. The cells and tissues were counterstained with DAPI to delineate the nuclei, and then the sections were examined by confocal microscopy (LSM-700; Carl Zeiss, Jena, Germany).

### Assessment of Renal Tissue Morphology

Four-micrometer-thick paraffin-embedded kidney tissues sections from the four mouse groups were dewaxed in xylene and rehydrated. To histologically assess tubule-interstitial injury and fibrosis, we stained the sections with periodic acid-Schiff (PAS) or Masson’s trichrome reagents. Twenty high-power fields of cortico-medullary areas in each section were randomly selected for analyses of tubule-interstitial injury and fibrosis and were blindly scored by an independent pathologist. Total tubular injury was graded on the following scale of 0 to 3, based on the percentage of normal tubules and the amounts of tubular dilatation and tubular epithelial cell destruction: 0, absent; 1, 1–25% of the tubular area is affected; 2, 26–50% of the tubular area is affected; and 3, >50% of the tubular area is affected^50^. Fibrosis was expressed as the percentage of positive area (twenty) in the total field using computer-assisted image analysis^51^.

### Electron Microscopy Analysis

Renal cortices were minced into 1-mm^3^ pieces and processed for electron microscopy. Thin sections were prepared for electron microscopy to delineate the extent of mitochondrial fragmentation in the tubules^41^. Mitochondria longer than 2 *μ*m were considered filamentous, and those shorter than 0.5 μm and with a spherical configuration were considered fragmented.

### Statistics

The data are expressed as the mean ± SEMs. Comparison between groups was made with one-way ANOVA, followed by the Student–Newman–Keuls test. P values < 0.05 were considered statistically significant. All of the analyses were completed using SPSS software (version 22; SPSS, Chicago, IL, USA) for Windows.

## Electronic supplementary material

Supplementary information
