# Supplementary material for: Chloroquine and amodiaquine enhance AMPK phosphorylation and improve mitochondrial fragmentation in diabetic tubulopathy
Source: Sci Rep. 2018 Jun 8;8:8774. doi: 10.1038/s41598-018-26858-8 (PMC5993726; doi:10.1038/s41598-018-26858-8)

Supplementary information.

Chloroquine and amodiaquine enhance AMPK phosphorylation and improve mitochondrial fragmentation in diabetic tubulopathy

(Chloroquine and amodiaquine have effects on diabetic tubulopathy)

Hye Yun Jeong1, Jun Mo Kang1, Hak Hoon Jun2, Dong-Jin Kim3, Seon Hwa Park3, MinJi Sung1, Jin Hyung Heo4, Dong Ho Yang1, Sang Ho Lee3*, So-Young Lee1*

1Division of Nephrology, Department of Internal Medicine, CHA Bundang Medical Center, CHA University, Seongnam, 2Department of Surgery, CHA Bundang Medical Center, CHA University, Seongnam, 3Division of Nephrology, Department of Internal Medicine, Kyung Hee University Hospital at Gangdong, Kyung Hee University, Seoul, 4Department of Pathology, CHA Bundang Medical Center, CHA University, Seongnam, South Korea

Corresponding Authors:

Dr. So-Young Lee, Division of Nephrology, Department of Internal Medicine, CHA University School of Medicine, CHA Bundang Medical Center, 59 Yatap-ro, Bundang-gu, Seongnam-si, 13496, South Korea. Phone: +82-31-780-5025, Fax: +82-31-780-5219, E-mail: ysy0119@cha.ac.kr

Or Dr. Sang Ho Lee, Division of Nephrology, Department of Internal Medicine Kyung Hee Univ. Hospital at Gangdong, 892 Sangil-ro, Gangdong-gu, Seoul, 05278, South Korea. Phone +82-1-440-6121, E-Mail: lshkidney@khu.ac.kr

H. Y Jung, J. M. Kang, H. H. Jun contributed equally to the work.

S-Y. Lee and S. H. Lee contributed equally to the work.

Supplementary Fig. S1. pAMPKα expression in hRPTCs under HG conditions. (a) Western blot analyses showed that pAMPKα expression decreased according to the time of incubation in 30 mM D-glucose. (b) pAMPKα expression in hRPTCs after incubation with 5 mM or 30 mM D-glucose or 25 mM mannitol (* $p < 0.05$ vs 5 mM D-glucose, #$p < 0.05$ vs 30 mM D-glucose). The original images of (c) pAMPKα and (d) GAPDH in Supplementary Fig. S1a. The original images of (e) pAMPKα and (f) GAPDH in Supplementary Fig.S1b.

Supplementary Fig. S2. CQ and AQ increased AMPKα phosphorylation in hRPTCs under HG conditions. (a) pAMPKα expression in hRPTCs under HG conditions after treatment with different concentrations of CQ. (b) pAMPKα expression in hRPTCs under HG conditions after treatment with different concentrations of AQ. The original images of (c) pAMPKα, (d) AMPKα and (e) GAPDH in Supplementary Fig. S2a. The original images of (f) pAMPKα, (g) GAPDH, (h) AMPKα and (i) GAPDH in Supplementary Fig. S2b.

Supplementary Fig. S3. CQ and AQ normalized the levels of AMP/ATP ratio in hRPTCs under HG condition. Results are means ± SEM. for experiments in triplicate. (*$p < 0.05$ vs 5 mM, #$p < 0.05$ vs 30 mM)

Supplementarly Fig. S4. CQ and AQ induce LKB1 phosphorylation under HG conditions (a) Western blot analyses showed that pLKB1 expression increased after treatment with CQ and AQ in hRPTCs subject to high glucose. The original images of (b) pLKB1, (c) LKB1, and (d) β-actin in Supplementary Fig S4. (**$p < 0.01$ vs 5 mM, #$p < 0.05$ vs 30 mM)

Supplementarly Fig. S5. Effects of CQ and AQ on the expression of pAMPKα and pPGC1α in diabetic kidneys. (a) CQ and AQ restored pAMPKα and pPGC1α expression in diabetic kidneys. The original images of (b) pAMPKα, (c) AMPKα, (d) pPGC1α, (e) PGC1α and (f) βactin in Supplementary Fig. S5.

Supplementary Fig. S6. Western blot of cell lysate in Fig.1. The original images of (a) pAMPKα, (b) AMPKα, and (c) pPGC1α in Fig. 1A. The original images of (d) PGC1α, (e) pPGC1α, and (f) βactin in Fig. 1B.

Supplementary Fig. S7. Western blot of cell lysate in Fig. 2. The original images of (a) pAMPKα, (b) AMPKα, (c) pPGC1α, (d) PGC1 α, and (e) βactin in Fig 2A. The original images of (f) pAMPKα, (g) AMPKα, (h) pPGC1α, (i) PGC1α, and (j) βactin in Fig. 2B.

Supplementary Fig. S8. Western blot of cell lysate in Fig. 3. The original images of (a) Tom20, and (b) GAPDH in Fig. 3B. The original images of (c) Drp1, (d) Mfn1, and (e) GAPDH in Fig. 3C.

Supplementary Fig. S9. Western blot of cell lysate in Fig. 4. The original images of (a) Tom20, (b) Drp1, (c) Mfn1 and (d) βactin in Fig. 4A. The original images of (e) Tom20, (f) Drp1, (g) Mfn1, and (h) βactin in Fig. 4B.

Supplementary Fig. S10. Western blot of cell lysate in Fig. 5. The original images of (a) Bcl-2, (b) Bax, and (c) GAPDH in Fig. 5B. The original images of (d) Cyt.C, and (e) GAPDH in Fig. 5C.

Supplementary Fig. S11. Western blot of cell lysate in Fig. 6. The original images of (a) TGF-β1, and (b) GAPDH in Fig. 6A. The original images of (c) E-cad, (d) α-SMA, (e) fibronectin and (f) GAPDH in Fig.6B.

Supplementary Fig. S12. Western blot of tissue lysate in Fig. 8. The original images of (a) Tom20 in Fig. 8A. The original images of (b) Drp1, and (c) Mfn1 in Fig. 8B. The original images of (d) βactin in Fig. 8A and 8B.

Supplementary Fig. S13. Western blot of tissue lysate in Fig. 9. The original images of (a) Bcl-2, (b) Bax, and (c) Cyt.C in Fig. 9A. The original images of (d) TGF-β1, (e) E-cad, (f) α-SMA , and (g) fibronectin in Fig. 9D. The original images of (h) βactin in Fig. 9A and 9D.

Supplementary Fig. S1.

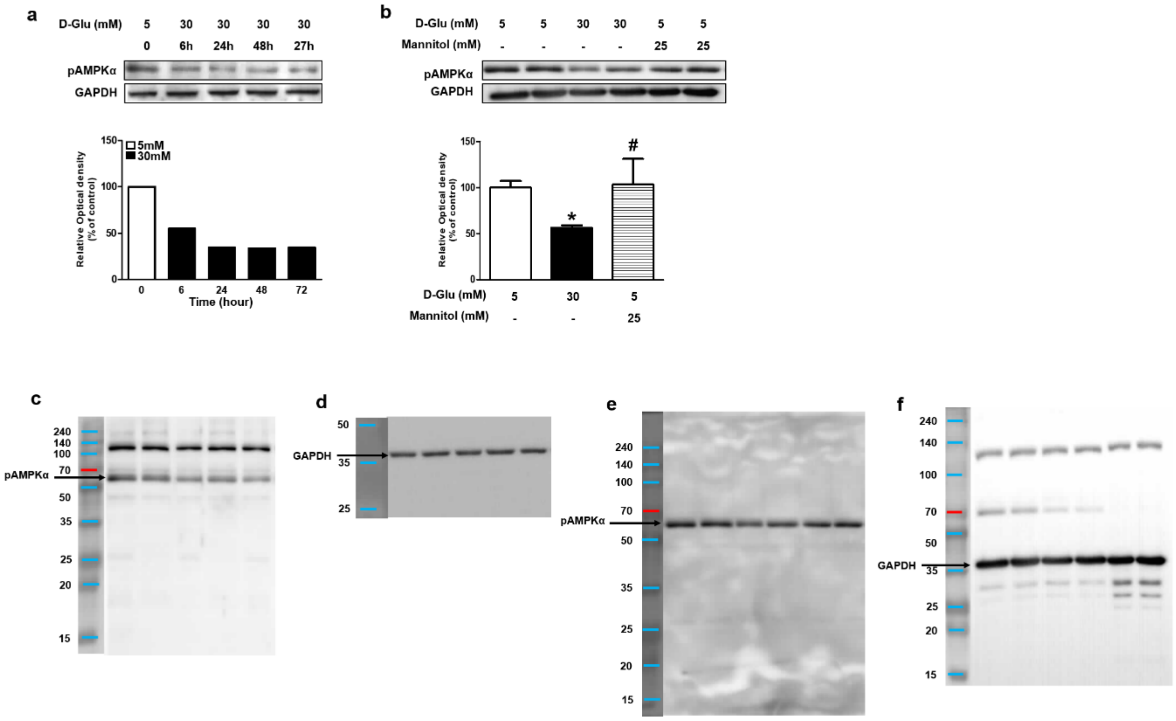

Supplementary Fig. S2.

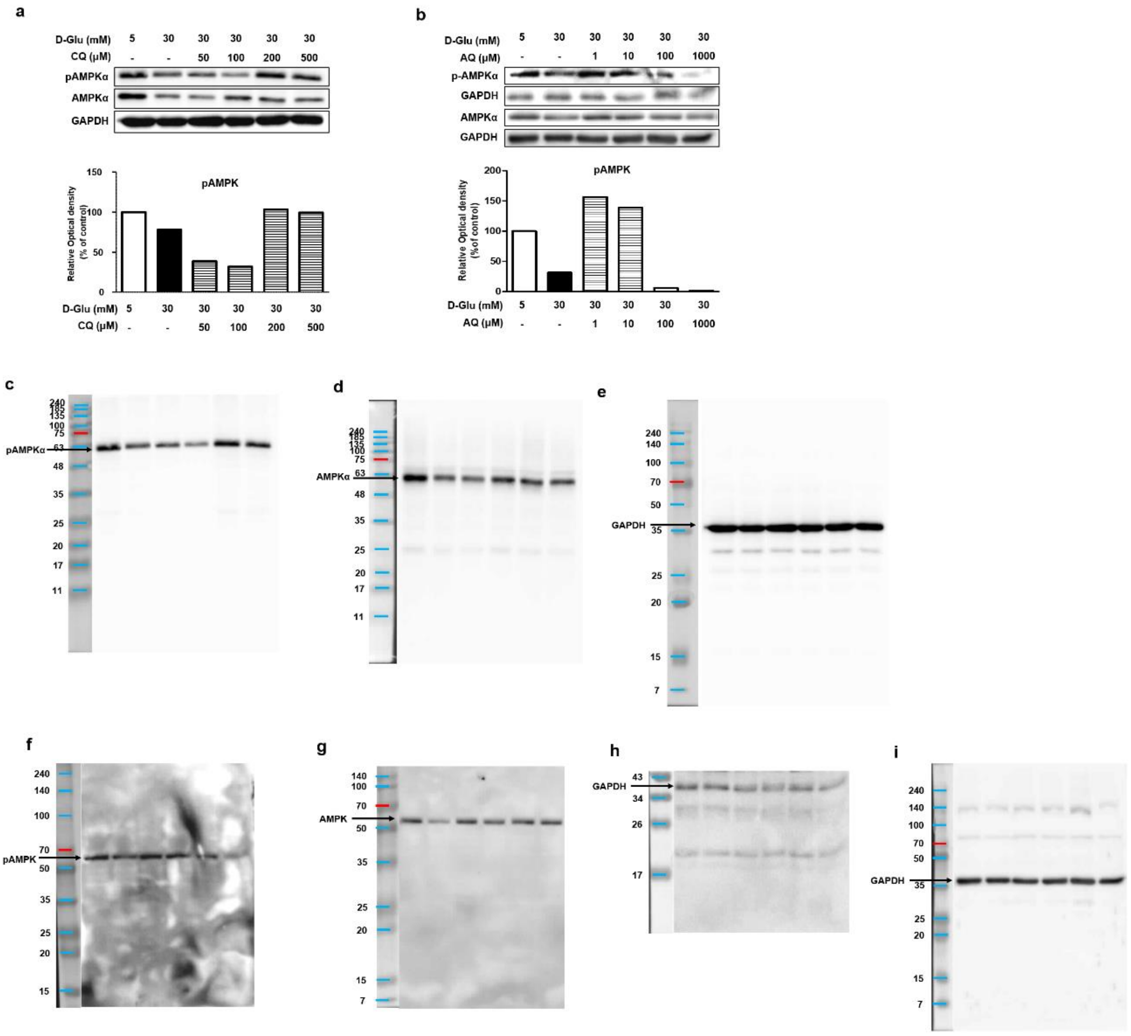

Supplementary Fig. S3.

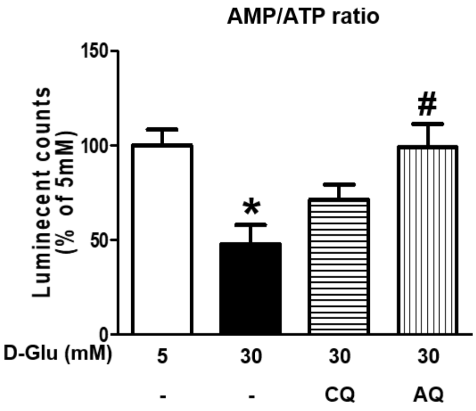

Supplementary Fig. S4.

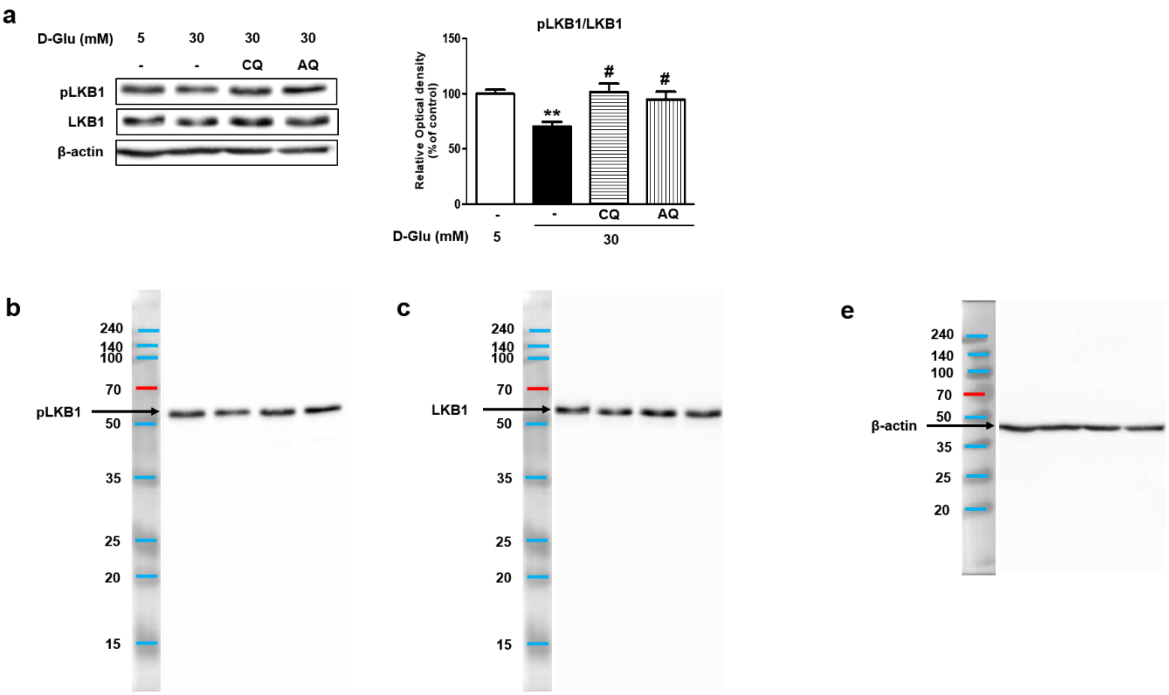

Supplementary Fig. S5.

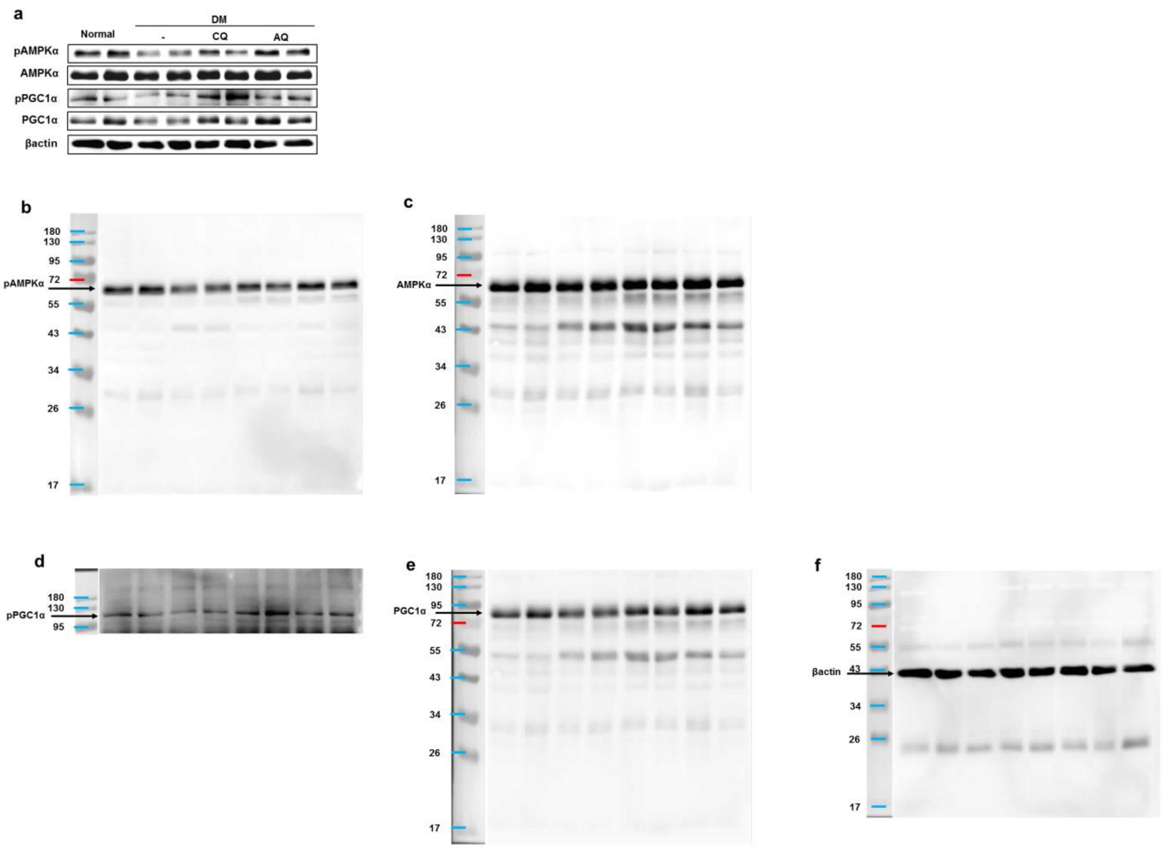

Supplementary Fig. S6.

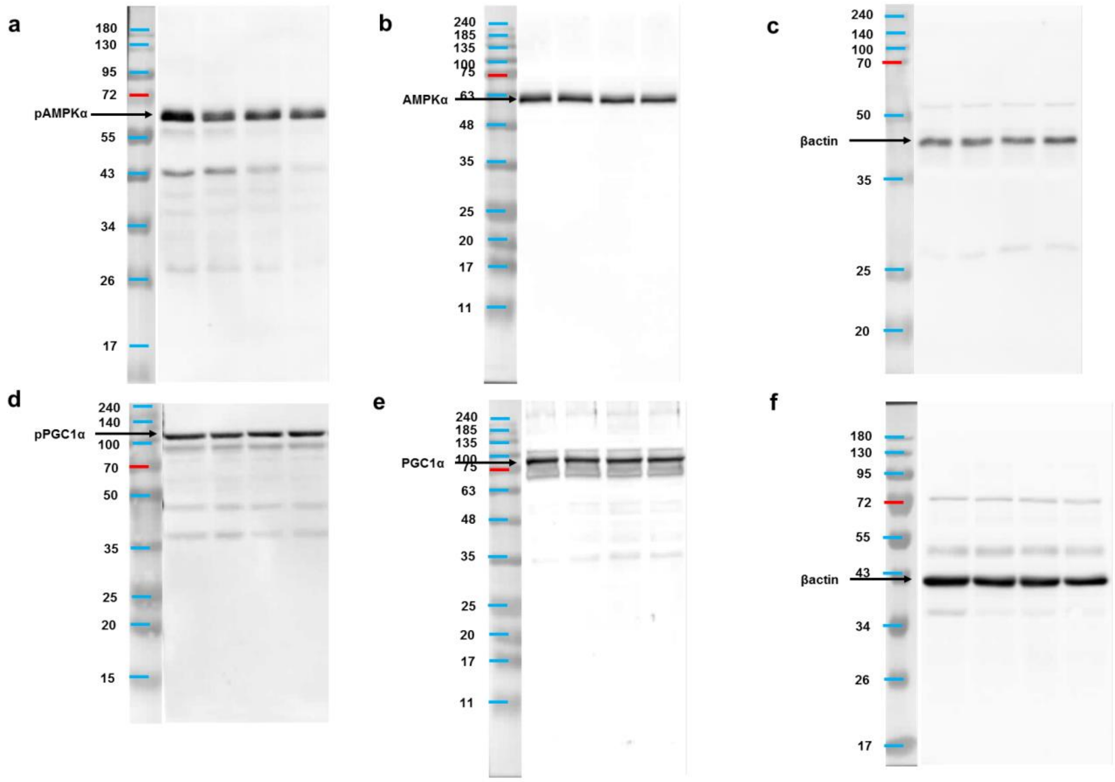

Supplementary Fig. S7.

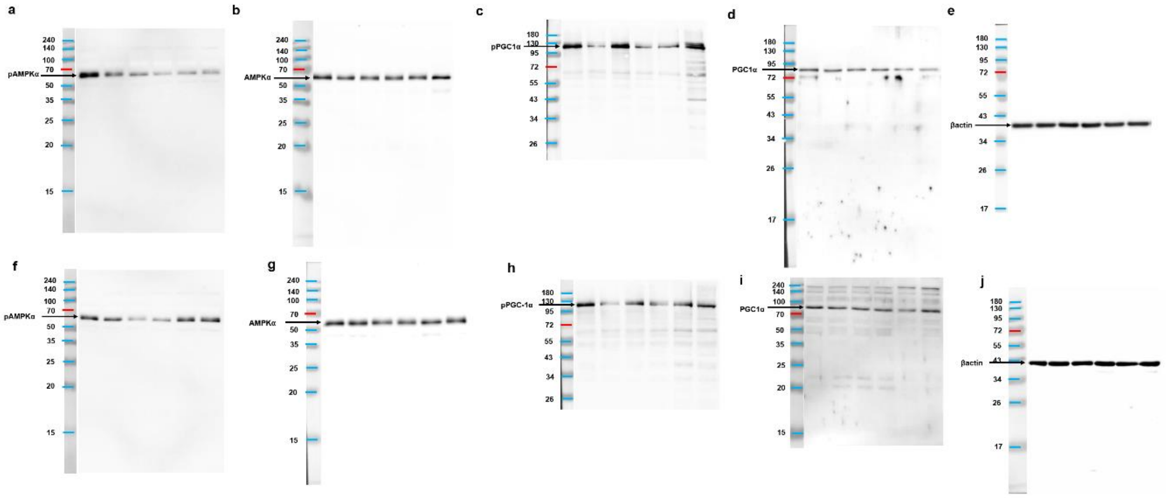

Supplementary Fig. S8

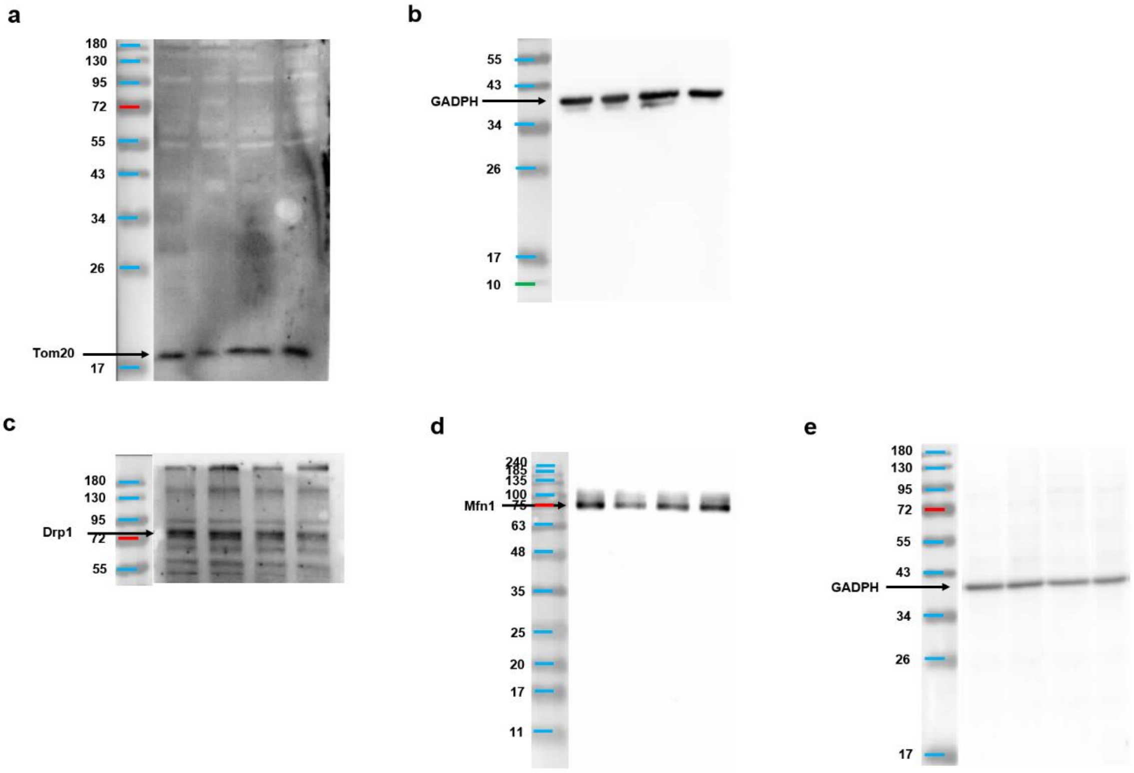

Supplementary Fig. S9

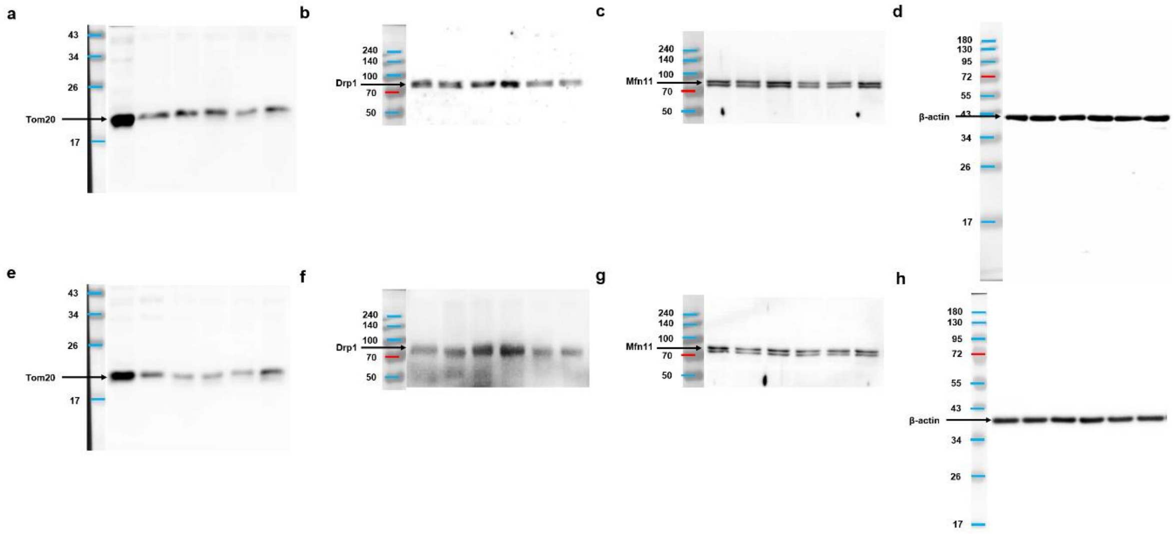

Supplementary Fig. S10.

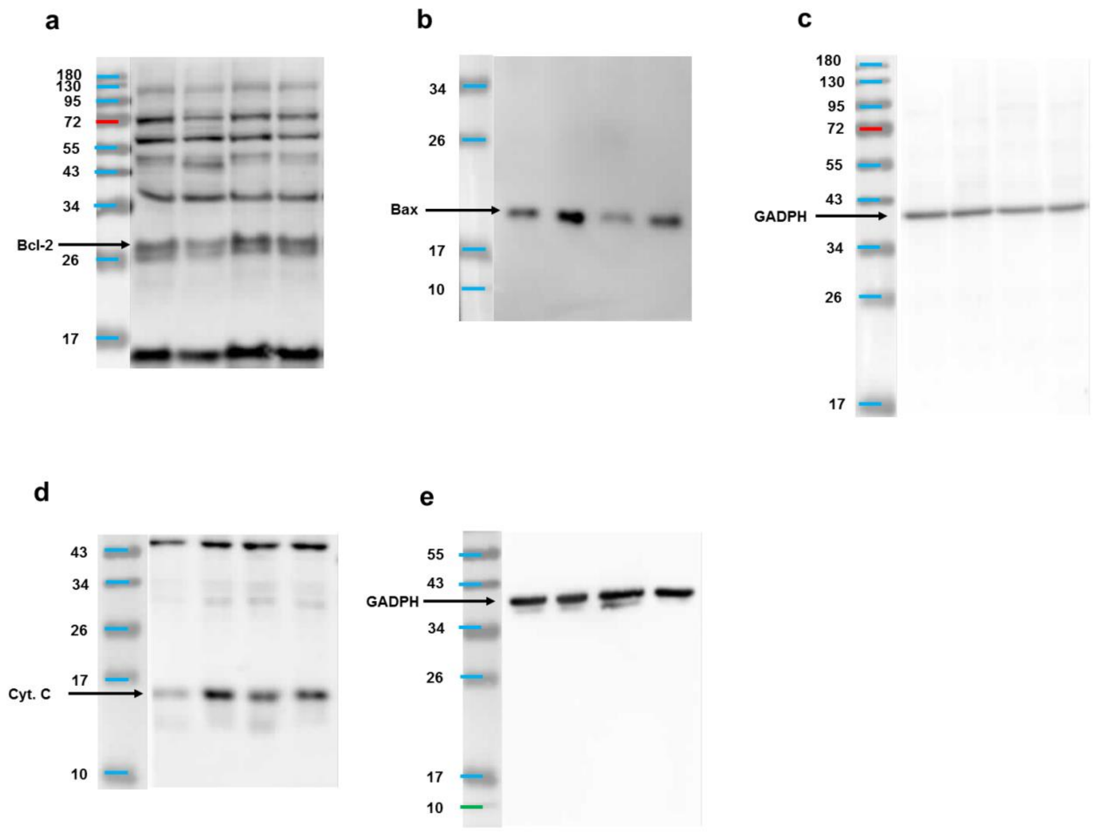

Supplementary Fig. S11

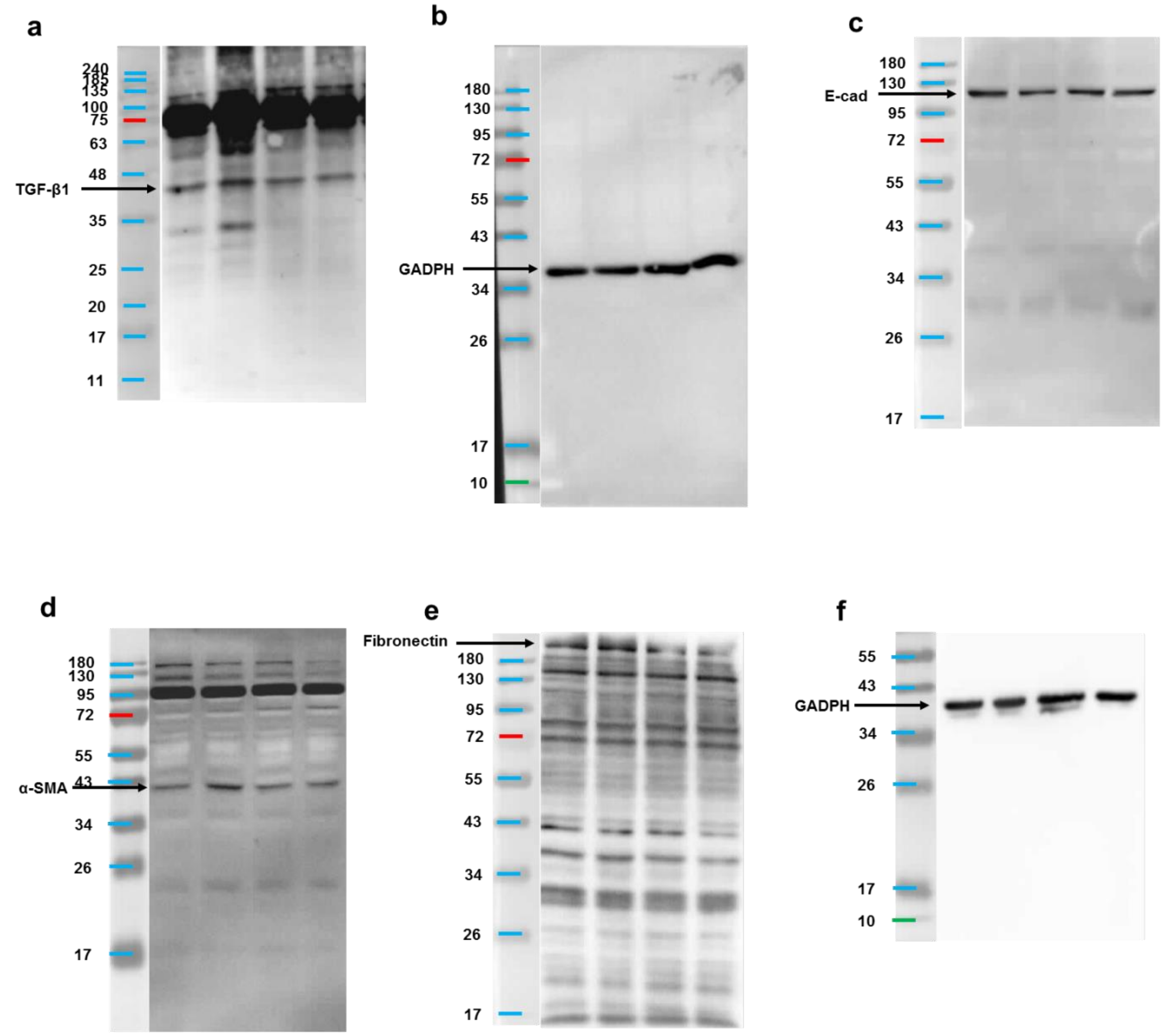

Supplementary Fig. S12

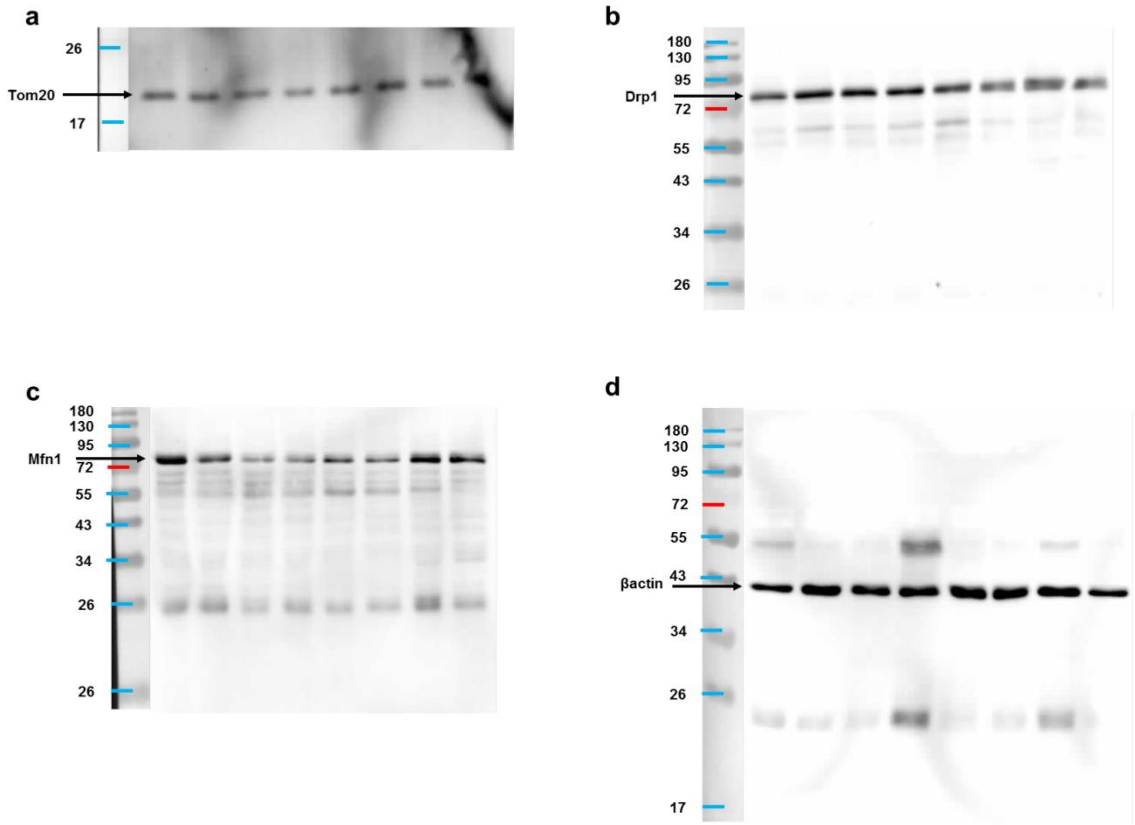

Supplementary Fig. S13.

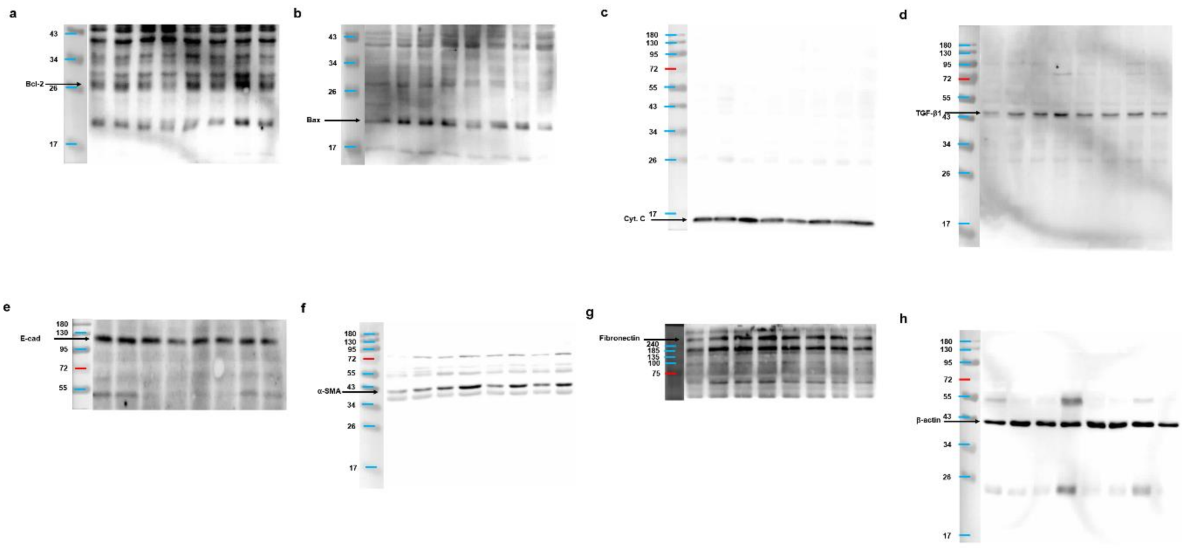

Supplement: Supplementary file 1 — Supplementary information [file 41598_2018_26858_MOESM1_ESM.pdf]
